# α-synuclein expression in glioblastoma restores tumor suppressor function and rescues temozolomide drug resistance

**DOI:** 10.1038/s41419-025-07509-z

**Published:** 2025-03-19

**Authors:** Eric Duplan, Aurore Bernardin, Thomas Goiran, Nathalie Leroudier, Mathew Casimiro, Richard Pestell, Shinya Tanaka, Celine Malleval, Jerome Honnorat, Ahmed Idbaih, Lucie Martin, Hélène Castel, Frédéric Checler, Cristine Alves da Costa

**Affiliations:** 1 University Côte d’azur, INSERM U1323, CNRS UMR7275, Institut de Pharmacologie Moléculaire et Cellulaire (IPMC), team labeled “Laboratory of Excellence (LABEX) Distalz”, 06560 Valbonne, France; 2 Department of Science and Mathematics, Abraham Baldwin Agricultural College, Tifton, GA 31794 USA; 3 Pennsylvania Cancer and Regenerative Medicine Research Center, Baruch S. Blumberg Institute, Pennsylvania Biotechnology Center, Wynnewood, PA 19096 USA; 4 The Wistar Institute, Philadelphia, PA 19107 USA; 5 Garvan Institute of Medical Research, and, St Vincent’s Clinical School, UNSW Sydney, 384 Victoria Street, Darlinghurst, NSW 2010 Australia; 6 Department of Cancer Pathology, Faculty of Medicine, and Institute for Chemical Reaction Design and Discovery (WPI-ICReDD), Hokkaido University, N15, W7, Sapporo, 060-8638 Japan; 7 Department of Neuro-Oncology, Hospices Civils de Lyon, Hôpital Neurologique, Institute MeLiS-UCBL-CNRS UMR 5284. INSERM U1314, University Claude Bernard Lyon 1, Lyon, 69008 France; 8 Sorbonne Université, AP-HP, Institut du Cerveau - Paris Brain Institute - ICM, Inserm, CNRS, Hôpitaux Universitaires La Pitié Salpêtrière - Charles Foix, DMU Neurosciences, Service de Neuro-Oncologie-Institut de Neurologie, F-75013 Paris, France; 9 Univ Rouen Normandie, Inserm U1245, Normandie Univ, F-76000 Rouen, France; 10 Institute of Research and Innovation in Biomedicine (IRIB), 76000 Rouen, France; 11 Cancer and Cognition Platform, Normandie Univ, 14000 Caen, France

**Keywords:** Cancer, Diseases

## Abstract

Several studies have shown that Parkinson’s disease causative gene products, including α-synuclein (α-syn), display tight links with the tumor suppressor p53. The purpose of this study is to determine the implication of α-syn in glioblastoma development and elucidate how it elicits a tumor suppressor function. We show that the expression of α-syn, a TP53 transcriptional target and a key molecular player in Parkinson’s disease, is detected in 1p/19q-codeleted and isocitrate dehydrogenase (IDH)-mutant oligodendroglioma and in IDH-wild-type glioblastoma, while reduced in glioblastoma biopsies, corroborating the link of α-syn expression with a better prognosis among all glioma patients. Accordingly, protein expression is drastically reduced in oligodendrogliomas and glioblastoma biopsies. This could be accounted for by a reduction of p53 transcriptional activity in these samples. Interestingly, genetic manipulation of p53 in glioblastoma cells and in mouse brain shows that p53 up-regulates α-synuclein, a phenotype fully abolished by the prominent p53 hot spot mutation R175H. Downstream to its p53-linked control, α-syn lowers cyclin D1 protein and mRNA levels and reduces glioblastoma cells proliferation in a cyclin D1-dependent-manner. Further, in temozolomide (TMZ)-resistant U87 cells, α-syn reduces O^6^-methylguanine-DNA methyltransferase (MGMT) expression and rescues drug sensitivity by a mechanism implying its transcriptional activation by X-box binding protein 1 (XBP1), an effector of the UPR response. Furthermore, α-syn lowers MGMT and cyclin D1 (CCDN1) expressions and reduces tumor development in allografted mice. Overall, our data reveals a new role of α-syn as an oligodendroglioma biomarker and as a glioblastoma tumor suppressor capable of either potentiate TMZ effect or avoid TMZ-associated resistance.

## Introduction

Parkinson’s disease (PD) and cancer are two major age-related health problems. Interestingly, epidemiological surveys have shown that their prevalence is inversely correlated. Thus, several studies revealed that PD-affected patients show a decreased risk of developing several cancer types [1, 2]. This inverse relationship is not unexpected when one considers that PD is clearly linked to an exacerbated p53-dependent cell death [3, 4] while conversely, in about half of cases, tumorigenicity is often due to impaired p53 function leading to increased proliferation. The latter phenomenon is often due to p53 hot spot mutations triggering the loss of its transcriptional function. Thus, these complex and mechanistically opposite pathologies could well be linked to the exacerbation/impairment of common cellular pathways and by consequence, could share one or many molecular denominators. In agreement with this hypothesis, it is interesting to note that several PD-causative gene products are abnormally expressed in several cancer types [5, 6] and that many of them can modulate and can be modulated by p53 [7–10].

Numerous reports documented that members of the synucleins family could be abnormally expressed in several cancers [11–13] and were paradoxically capable of modulating either proliferative or apoptotic signaling pathways. α-synuclein (α-syn) is a small aggregation-prone protein, the toxic role of which has been extensively linked to PD etiology [14, 15]. Thus in most of sporadic and genetic PD cases [16], α-syn aggregates fill intracellular inclusions named Lewy bodies that correspond to the canonical anatomical PD stigmata. We previously showed that there existed a functional interplay between α-syn and p53. Thus, in neuronal cells, we demonstrated that α-syn down-regulates p53 which, in turn controls positively the transcription/expression of α-syn. This feedback process is likely responsible for the control of a physiological homeostasis in a healthy cellular context [7, 17]. This led us to postulate that p53 dysfunction in cancer cells could impair this physiological interplay and favor brain tumor development via the deregulation of α-syn-mediated cell death/proliferation control.

Our data identify α-syn as a good prognosis marker of glioma and as an early marker of glioma progression. Importantly, it demonstrates that α-syn per se modulates cell proliferation in a cyclin D1-dependent manner. Further, we show that TMZ increases α-syn protein and mRNA expressions but reduces CCDN1 expression, in agreement with a cascade by which TMZ could occur upstream to α-syn-mediated reduction of CCDN1 in GBM. This appears to be linked to the ability of TMZ to up-regulate the ER-stress mediator XBP1S (the spliced and active form of XBP1) which, we show to act as a transcriptional activator of α-syn. Of utmost importance, α-syn expression lowers MGMT expression and partly rescues U87 TMZ-induced resistance. Accordingly, orthotopic injection of GL261 cells expressing α-syn in mice evidenced an α-syn tumor suppressor function in vivo.

Overall, we delineate a new molecular cascade indicating that the disruption of p53-α-syn feedback loop homeostasis may lead to a critical shutdown of α-syn tumor suppressor function and favor the development of brain tumors. Furthermore, this study proposes α-syn as a putative agent able to, at least partly, rescue TMZ-induced resistance explaining most of cerebral cancer relapse.

## Materials and methods

### In silico SNCA expression analysis in gliomas from available public databases

The detailed description of in silico SNCA expression analysis in gliomas from available public databases; (https://singlecell.broadinstitute.org/single_cell/study/SCP393/single-cell-rna-seq-of-adult-and-pediatric-glioblastoma) according to the workflow described Fig. S1 and described in Supplementary Methods.

### Cellular models and pharmacological treatments

Description of cell lines, method of authentication and pharmacological treatments are described in Supplementary Methods (Cellular models and pharmacological treatments paragraph).

### Plasmid constructs

Sub-cloning of human wild-type p53 (flag-tagged) or its R175H mutant in the pcDNA3.1 (+) vector has been described [10]. The cloning of the 5’UTR of “human *SNCA* promoter” and its mutagenesis are described in the Supplementary Methods. The vector pA3-1745 CD1 Luc containing human CCND1 promoter region (portion −1745 to +134) was generated by one of us and described in [18]. The subcloning of human α-syn and mouse XBP1S coding sequences in the FUW lentiviral vector and virus production procedures are provided in Supplementary Methods.

### Caspase-3 activity measurements

Briefly, 8MG and U87 glioma cell lines stably expressing α-syn or an empty vector were grown in 6-well plates or 24 h. Caspase-3 activity was fluorimetrically recorded on a spectral scanning multimode reader (Varioscan, Thermo Fisher Scientific) as extensively described in [19].

### Proliferation assays

The impact of α-syn overexpression on cells proliferation was established by means of the xCELLigence impedance-based label-free real-time cellular analysis system (Acea Biosciences, Inc) and flow cytometry as described in [20]. Glioma cell lines over-expressing α-syn (by mean of transduction with FUW control or FUW α-syn viruses) were seeded at a density of 10000 cells in equilibrated E-plates and inserted in the xCELLigence analyzer.

### Luciferase-based reporter assays

Methodology linked to the analysis of p53 (*TP53*) transcriptional activity and cyclin D1 (*CCND1*), *SNCA* and *XBP1* promoter activities have been described in Supplementary Methods.

### Protein analysis by Western-blot

α-syn, CCND1, MGMT, p53, XBP1S, actin and tubulin expressions were analyzed in cell lines, mice brain homogenates and human biopsies as fully described in Supplementary Methods. All full gels are provided in as supplementary Material.

### RNA extraction, reverse transcription and real-time PCR analysis

*RNAs* from cells and mice brains were extracted and treated with DNAse using RNeasy or RNeasy Plus Universal Mini kits respectively following manufacturer’s instructions (Qiagen), reverse transcribed and analyzed by real-time quantitative PCR as described in Supplementary Methods.

### Allografts studies and in vivo transfection

Experimental procedures were carried out in accordance with the Directive 2010/63/EU directive 2010/63/EU of the European parliament and of the council of 22 September 2010 after approval of the use of animals for experimental purposes by the local French Ethics.

The detailed procedure is described in Supplementary Methods.

### Tumor volume analysis

Tumor volume quantification procedure is detailed in Supplementary Methods. The maximal tumor size (200 mm3) permitted by French ethics committee was not exceeded.

### Patient’s biopsies cohort

The cohort used in this study has been characterized concerning the mutational status of p53 and extensively described in [10]. A brief description is provided in Supplementary Methods.

### SNCA genomic BAC ARRAY-CGH and TP53 mutation analysis

BAC ARRAY-CGH and TP53 mutation analysis are fully described in [10] and Supplementary Methods.

## Results

### α-synuclein expression varies according to IDH mutational status and 1p/19q deletion in gliomas

**Fig. 1:**
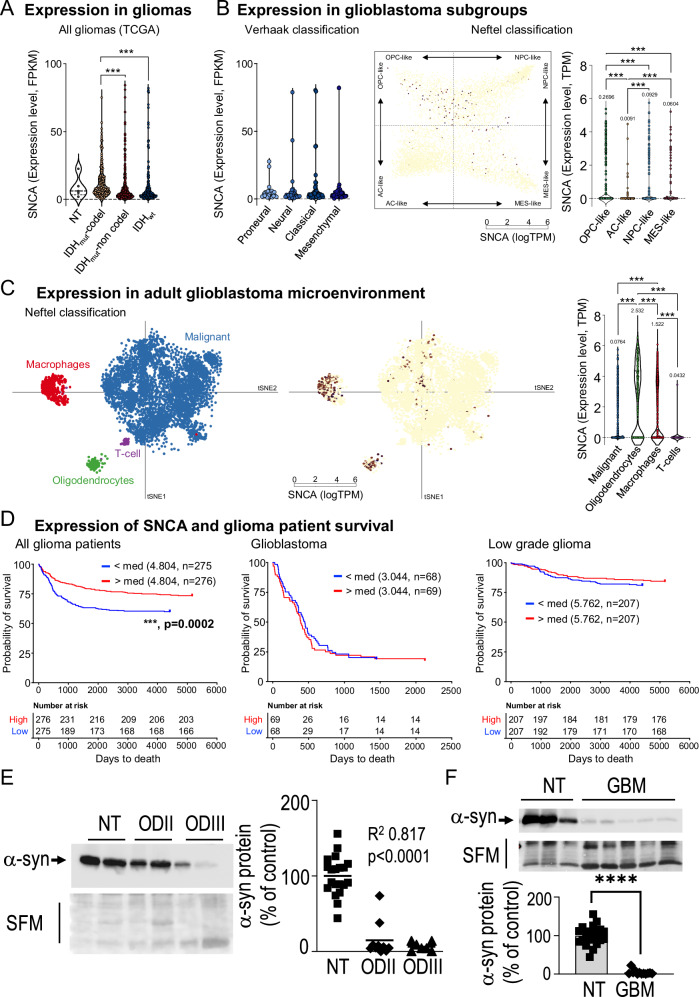
α-synuclein levels are reduced in human brain glioma. **A** SNCA expression in all IDH_mut_-codel, IDH_mut_-non codel et IDH_wt_ glioma samples from TCGA database. Violin plots of SNCA expression (FPKM, Fragment Per Kilobase Million) from RNA-seq analysis (***, P < 0.001; NT, non tumoral). **B** Left, SNCA expression (from TCGA) levels in Proneural, Neural, Classical and Mesenchymal glioblastoma (GBM) subtypes. Right, SNCA expression dispersion among the subgroups according to Neftel classification: oligodendroglial progenitor cell-like (OPC-like); astrocyte-like (AC-like); neural progenitor cell-like (NPC-like) and mesenchymal-like (MES-like). Each quadrant of the 2D representation (**B**, middle) corresponds to one cell state of Neftel classification. The exact position of adult GBM single cells (dots, n = 4916) indicates their relative score for the meta-modules and their color illustrates the expression level of SNCA (***, P < 0.001). Values above the violin corresponds to the mean expression in each subgroup. **C** Kaplan-Meier statistical analysis of the prognostic significance of SNCA in all gliomas (right), in GBM (middle) and in low grade glioma (right) patients. Blue lines represent low expression ( < median, (med)) and red lines represent high expression ( > median); *n* indicates number of samples in each low or high expression groups. (***, P = 0.0002). **D** Single cell RNA-seq (n = 5742) from Neftel classification by means of the t-distributed stochastic neighbor embedding (tSNE) plot, showing clustered cells based on the presence of chromosomal copy number aberrations (blue), or high expression of marker genes for macrophages (red), oligodendrocytes (green) or T-cell (purple). According to that clustering, color dots indicate SNCA expression levels (logTPM, ***, P < 0.001). Representative immunoblots and quantification of α-syn expression in non-tumoral (NT), grade II (N = 12) and III (N = 7) oligodendroglioma (**E**, One-way ANOVA, correlation trend analysis) or GBM biopsies (**F**, N = 12, Mann–Whitney test). Stain Free gel quantification method was used to normalize protein load charges. Bars represent the means ± SEM of α-syn protein expression as percent of control (NT biopsies) taken as 100. ****, P < 0.0001.

Two experimental set of data led us to consider that an α-syn-p53 functional interplay could contribute to brain tumor development. First, p53 is a key protein, the reduced expression of which has been consistently shown to be linked to glioma genesis [21]; second, we previously established that *SNCA* gene coding for α-syn behaves as a transcriptional target of p53 [17]. Thus, we speculated that a p53-dependent control of α-syn could well account, at least in part, to glioblastoma development. To investigate this hypothesis, we first performed an in silico bioinformatic study (see scheme in Fig. S1) examining the RNAseq and scRNAseq OMIC open-source data (see Material and Methods). The TCGA database (see Supplementary Methods) established the status of mRNA *SNCA* levels encoding α-syn in gliomas, according to their genetic classification. Thus, gliomas were separated into three classical groups according to the mutational status of the isocitrate dehydrogenase (IDH_wt_) wild-type or mutated (IDH_mut_), the latter associated or not with the 1p/19q codeletion (IDH-_mut_ codel or IDH-_mut_ non codel, respectively). *SNCA* mRNA levels were significantly lower in IDH-_mut_-non codel and IDH_wt_ gliomas than in IDH_mut_-codel samples (Fig. 1A). We also established the levels of *SNCA* mRNA according to the Verhaak classification [22] that discriminates pro-neural, neural, classical and mesenchymal GBM. No significant difference for *SNCA* mRNA could be observed between subtypes of GBM (Fig. 1B). To further characterize whether α-syn may be expressed in different subpopulations of GBM cells, according to neural cell lineage signature, we analyzed the public RNAseq database from 21 adult IDH_wt_ GBM and 7 pediatric high-grade gliomas encompassing 24,131 total cells. Here, only GBM cells were kept for the analysis, and as established by Neftel et al. [23], the tumor specimens clustered into four broad lineage categories, i.e malignant cells, oligodendrocytes, T cells, macrophages according to multi-gene expression signatures [23] (Fig. 1B). *SNCA* mRNA were found significantly more expressed in oligodendrocyte progenitor-like (OPC-like) subgroup compared with astrocyte-like (AC-like), neural progenitor like (NPC-like) and mesenchymal-like (MES-like) subgroups (P < 0.001) within malignant GBM cells (Fig. 1B, right panels), and while detected in macrophages, higher levels of *SNCA* were detected in normal oligodendrocytes than in malignant cells, macrophages and T-cells (Fig. 1C, P < 0.001). This agrees with data on *SNCA* expression retrieved from GBMseq databases showing higher expression in neurons, myeloid cells, oligodendrocytes and astrocytes, with almost undetectable level in neoplastic cells (Fig. S2B). Interestingly, by considering the spatial distribution of the different cell populations within a GBM, we analyzed *SNCA* expression from the IVY-GAP database, and showed that *SNCA* mRNA is detected in the infiltrating tumor and leading edge of the tumor (Fig. S2C), suggesting, based on the GTEx high expression of SNCA mRNA in brain (Fig. S2D), a role of α-syn within the brain microenvironment.

The above set of genetic and cytological data was strengthened by examining the link between the status of *SNCA* mRNA and glioma-affected patients’ survival. The expression of *SNCA* mRNA was increased in all gliomas-patients survival but not glioblastoma or low-grade glioma-affected patients (Fig. 1D, left panel). Overall, this data corroborated well with the better prognosis observed in IDHmut-codel glioma compared with IDHmut-non codel glioma patients (Fig. S2A).

### α-synuclein expression is reduced in tumor patients’ biopsies

Next, we examined the protein levels of α-syn in biopsies of brain tumor patients. We took advantage of a fully genetically characterized cohort of 26 glioma samples of different histology (15 oligodendrogliomas (OD), 11 glioblastomas (GBM)) and 20 epilepsy-derived surgery controls (NT) [10] to evaluate the expression of α-syn. Prior to this analysis, we first ruled out the possibility of a genetic-linked (*SNCA* gene amplification and/or deletion) modulation of α-syn in our cohort by means of *SNCA* genomic array characterization. Figure 1E, F shows that α-syn expression is reduced according to tumor grade in oligodendrocytomas (ODII (N = 10) or ODIII (N = 7), Fig. 1E) and GBM biopsies (Fig. 1F). Overall, this reduction of α-syn protein expression agrees with the decrease of α-syn mRNA levels observed in the TGCA database, thus supporting our initial hypothesis on a putative reduced tumor suppression role of α-syn in glioblastoma.

### Reduction of α-synuclein expression in GBM is due to p53 loss of function

**Fig. 2:**
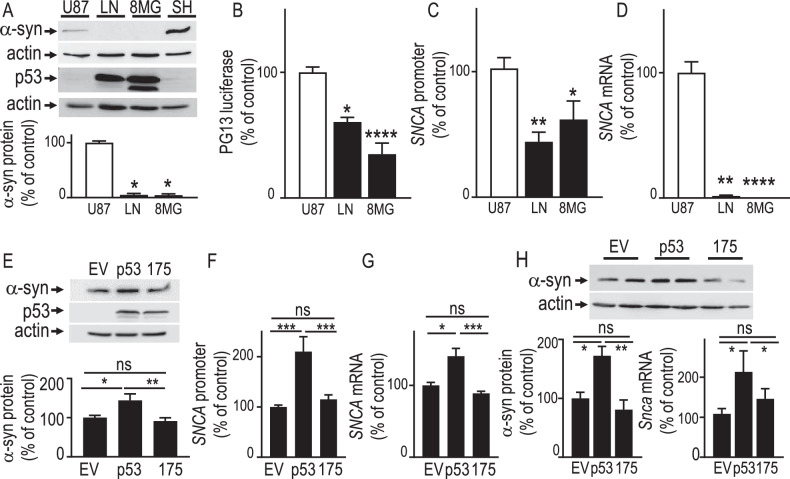
p53 regulates α-synuclein in vitro and in vivo. p53 protein expression (**A**), pG13-Luciférase reporter gene transactivation efficiency (**B**, N = 6, Kruskal Wallis ANOVA, Dunn’s multiple comparison post-test), α-syn protein expression (**A**, N = 6, Kruskal Wallis ANOVA, Dunn’s multiple comparison post-test), *SNCA* promoter activity (**C**, N = 8, One-way ANOVA, Sidak’s multiple comparison post-test) and *SNCA* mRNA levels (**D**, Kruskal Wallis ANOVA, Dunn’s multiple comparison post-test) in glioblastoma (U87, LN 215 or 8MG) as described in “Materials and Methods”. In (**A**), neuroblastoma (SH-SY5Y, SH) control cell line expressing high levels of α-syn. Bars are expressed as percent of control U87 cells and are the means ± SEM of 3 independent determinations performed in duplicates *, P < 0.05; **, P < 0.01; **** P < 0.001. Effect of empty vector (EV), wild-type (p53) and mutated p53 (R175H) cDNA transfection on α-syn protein expression (**E**, N = 9, One-way ANOVA, Sidak’s multiple comparison post test), *SNCA* promoter activity (**F**, N = 12, One-way ANOVA, Sidak’s multiple comparison post-test) and mRNA (**G**, N = 9, Kruskal Wallis ANOVA, Dunn’s multiple comparison post-test) levels in U87 cells. Values are expressed as percent of control (EV-transfected U87 cells) taken as 100 and are the means ± SEM of 3–4 independent determinations performed in triplicates. (*, P < 0.05; **, P < 0.01; *** P < 0.001). **H** α-syn protein levels (N = 7, Kruskal Wallis ANOVA, Dunn’s multiple comparison post-test) in mice brains injected with control (EV), wild-type (p53) or R175H mutated (175) p53 containing constructs as described in Materials and Methods. Bars represent the means ± SEM of α-syn protein expressed as percent of control EV transfected brains (taken as 100). (*, P < 0.05; **, P < 0.01). Actin protein levels are used as protein charge loading control in (**A**, **E**, **H**). p53 transfection efficacy is provided in Supplementary Fig. S2.

In order to gain insight into a role for p53 in α-syn expression in human gliomas, we examined α-syn protein, promoter activity and mRNA levels in SH-SY5Y neuroblastoma and several GBM cell lines harboring either low levels of wild-type p53 (U87 cell line) or high expression of transcriptionally inactive p53 mutants (LN215 and 8MG GBM cell lines). Figure 2A (N = 6) shows that α-syn is expressed in U87 cells harboring wild-type p53 and absent in LN215 and 8MG glioblastoma, thus supporting data in Fig. 1D indicating that α-syn is expressed in neurons but poorly detectable in GBM neoplastic cells. The analysis of p53 transcriptional activity by means of the PG13 TP53 reporter construct indicated that LN and 8MG cells were partially defective for p53 transcriptional activity (Fig. 2B). Of note, *SNCA* promoter activity (Fig. 2C) was reduced in p53-deficient cells (compare U87 cells with LN/8MG cell lines in Fig. 2C). This observation is confirmed at the α-syn mRNA levels that were poorly detectable in LN/8MG GBM cells (Fig. 2D).

### Cancer-linked p53 hot spot mutations abolish p53-dependent transcriptional regulation of α-synuclein, ex-vivo and in vivo

Glioma genesis is often linked to the loss of p53 transcriptional function due to hot spot mutations [24, 25]. Therefore, these mutations are useful means to demonstrate a direct link between the phenotypic concomitant reductions in p53 transcriptional function and α-syn mRNA and protein levels. As predicted transient overexpression of wild-type p53 in U87 cells increased α-syn protein expression (Fig. 2E), promoter activity (Fig. 2F) and mRNA levels (Fig. 2G). Interestingly, the hot spot mutation R175H, known to inactivate p53 DNA-binding properties, fully abolished p53-mediated α-syn/SNCA protein and gene regulation (Fig. 2E–G) in U87 cells. Next, we examined the influence of the wild-type or R175H p53 on α-syn expression in mice brain upon an in vivo transfection approach. Figure 2H shows that equal overexpression (see p53 mRNA levels Fig. S3) of wild-type and mutated p53 leads to an increase of α-syn protein levels, that is abolished by the R175H hot spot mutation.

### α-synuclein triggers a tumor suppressor-like phenotype in GBM cells

**Fig. 3:**
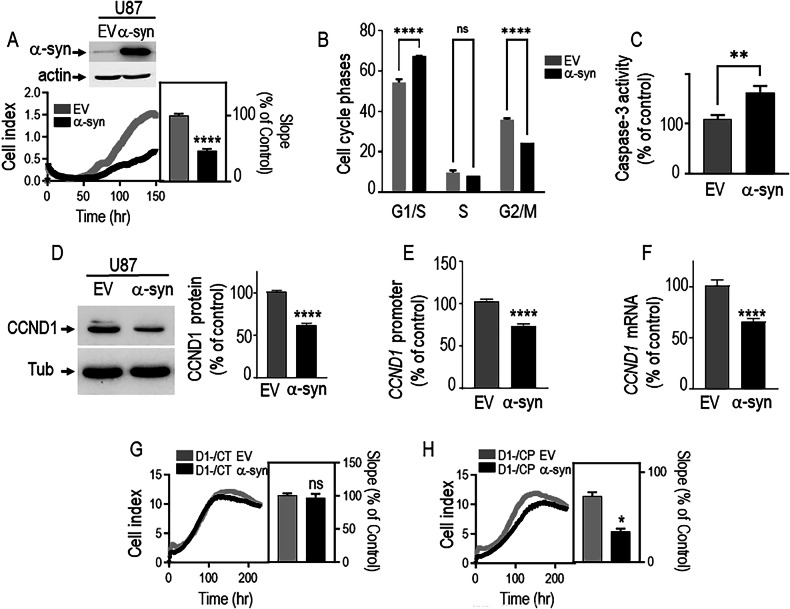
α-Synuclein down-regulates GBM cells proliferation via the regulation of cyclin D1. **A** Stable lentiviral overexpression of α-syn (**A**, upper) in U87 cells and influence on proliferation assessed by impedance-based approach (N = 12, impaired *t* test). Actin expression is provided as a control of protein load. Histogram illustrates the slopes of the proliferation curves expressed as percent of Fuw (EV) control cells slope and correspond to the means ± SEM of 4 independent experiments performed in triplicates. ****, P < 0.0001. **B** Impact of a-syn in U87 cell cycle dynamics by FACS analysis as described in methods. Data are expressed as percent of EV control cells taken and are the means ± SEM of 2 independent experiments performed in triplicates. ****, P < 0.0001 and ns, non-statistically significant. **C**–**F** Caspase 3 activity, cyclin D1 (CCND1) protein expression (**C**), promoter activation (**D**) and mRNA levels (**E**) (measured as described in “Methods”) in EV- and α-syn-infected U87 cells (N = 15 impaired t test). Data are expressed as percent of EV control cells (taken as 100%) and are the means ± SEM of 5 independent experiments performed in triplicates. **, P < 0.01; ****, P < 0.0001. Impact of EV or α-syn on the proliferation of 3T3 cells either depleted of cyclin D1 (D1-/CT EV and D1-/CT α-syn) (**G**, N = 9, unpaired t test) or rescued for cyclin D1 (D1-/CP EV and D1-/CP α-syn) (**H**, N = 4, Mann–Whitney test). Quantification analyses of the slopes of the curves in G and H correspond to the means ± SEM of 2–3 independent experiments performed in triplicates (**G**) or duplicates (**H**). *, P < 0.05; ns, non-statistically significant.

In order to determine if the reduction in α-syn in tumor cells and in biopsies contributes to the tumorigenesis process we examined the effect of α-syn rescue on U87 GBM cell proliferation using an impedance-based label-free real-time analysis. Cell index profiles (curves in Fig. 3A) and slope curves quantification (histogram in Fig. 3A) indicate that α-syn overexpression (see α-syn expression in Fig. 3A upper) decreases U87 cell proliferation. This data was corroborated by FACS analysis (Fig. 3B) showing that α-syn overexpression triggers a blockage of cells proliferation in G1/S phase, suggesting the implication of the key G1/S modulator, cyclin D1, in α-syn mediated U87 anti-proliferative effect. Then, we examined whether the above-described α-syn-linked anti-proliferative phenotype could be associated with activity of the cell death mediator caspase-3. Indeed, α-syn expression elevated caspase-3 activity in basal (Fig. 3C) conditions.

### α-Synuclein mediates decreased cell proliferation in GBM cells via the transcriptional regulation of cyclin D1

Next, to gain insights on the mechanisms by which α-syn could control GBM cell proliferation, we examined the impact of α-syn on cyclin D1(CCND1), a key regulator of G1/S phase cell cycle transition [26]. This choice was motivated by the recent work of Jia et al showing an upregulation of cyclin D1 by α-syn in neurons [27]. Figure 3D–F shows that in U87 cells, in contrast to neuronal cells, α-syn overexpression triggers a significant reduction of CCND1 protein (Fig. 3D), promoter activity (Fig. 3E) and mRNA levels (Fig. 3F), suggesting that α-syn-mediated control of proliferation could occur, at least in part, through the modulation of *CCND1* transcription and is dependent of the cellular physiopathological context.

We used a cyclin D1^−/−^ cellular model either rescued (D1-/CP) or not (D1-/CT) for cyclin D1 (see expressions Fig. S4A) to evaluate the contribution of CCND1 in α-syn mediated control of cellular proliferation. We first observed that slope (% of control) reflecting cell proliferation rate was higher in D1-/CP than in D1-/CT cells (Fig. S4B) confirming that cyclin D1 exacerbates cell proliferation. Interestingly, α-syn overexpression in both cells (see expressions in Fig. S4B,C) indicated that α-syn was unable to reduce cellular proliferation in the absence of endogenous cyclin D1 (compare D1-/CT EV and D1-/CT α syn in Fig. 3G) while cell proliferation was reduced by α-syn in cells in which CCND1 deficiency was genetically rescued (compare D1-/CP EV and D1-CT/α-syn in Fig. 3H). This data indicates that α-syn controls GBM proliferation in a CCND1-dependent manner.

### α-Syn stands downstream of p53 in the control of GBM cell proliferation

α-syn can control p53 levels in a neuronal context [7] and p53 itself can control cyclin D1 transcription [28]. This questions if α-syn controls CCND1 upstream or downstream p53. If α-syn occurs upstream to p53, p53 deficiency should abolish α-syn-mediated control of GBM cells proliferation. To address this question, we took advantage of a GBM cell model (8MG) in which p53 is transcriptionally inactive. Figure S5A, B clearly demonstrates that α-syn still downregulates proliferation (Fig. S54A) and induces the expression of the effector caspase 3 (Fig. S5B) in 8MG cells. Moreover, in agreement with our data, α-syn lowers cyclin D1 protein expression (Fig. S5C), promoter transactivation (Figure S5D) and mRNA levels (Fig. S5E). Overall, this data indicates that α-syn decreases cell proliferation independently of p53 and downstream to it, at least in part via the control of cyclin D1 transcription.

### Evaluation of the therapeutic potential of α-syn in GBM cells

**Fig. 4:**
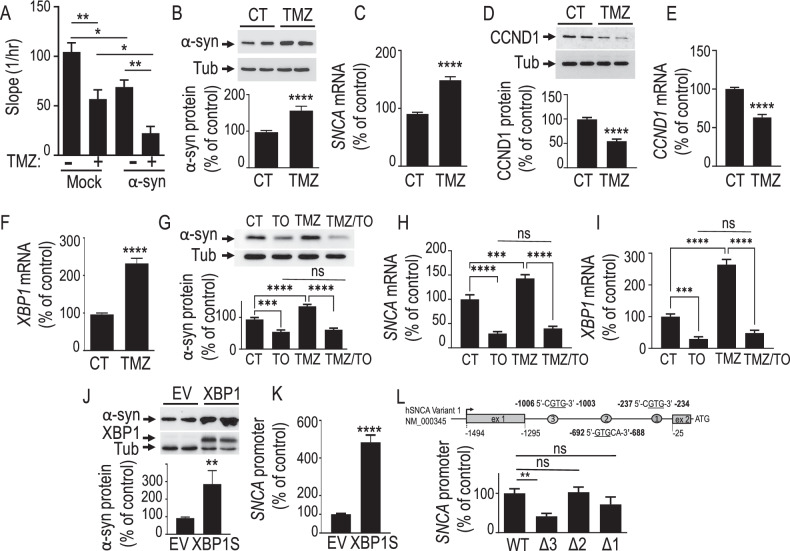
Temozolomide up-regulates α-syn and potentiates its anti-proliferative phenotype via XBP1S-mediated ER stress induction. **A** N = 6, One-way ANOVA, Sidak’s multiple comparison post-test, additive effects of temozolomide treatment (TMZ, 100 µM, 48 h) and α-syn stable expression on U87 cells proliferation. The bars represent means ± SEM of 3 independent experiments done in duplicates. *, P < 0.05; **, P < 0.01. U87 control cells were treated with Temozolomide (TMZ, 100 µM, 48 h) or vehicle (CT) then α-syn protein (**B**, N = 15, Mann–Whitney test), *SNCA* mRNA (**C**, N = 15, unpaired t test), cyclin D1 (CCND1) protein (**D**, N = 12, unpaired t test), *CCND1* mRNA (**E**, N = 12, unpaired t test) and *XBP1S* mRNA (**F**, N = 12, impaired t test) expressions were assessed as described in “Materials and Methods”. Values are expressed as percent of vehicle-treated control cells (CT) taken as 100 and represent the means ± SEM of 4–5 independent experiments performed in triplicates. ****, P < 0.0001. α-syn protein (**G**, N = 9, Mann Whitney test), *SNCA* mRNA (**H**, N = 9, Mann–Whitney test) and *XBP1S* mRNA (**I**, N = 9, One-way ANOVA, Sidak’s multiple comparison test) expressions were analyzed in U87 control cells treated with either temozolomide (TMZ, 100 µM, 48 h), toyocamycin (TO, 1 µM, 24 h) both TMZ and TO or with the appropriate vehicle (CT). Upper panel in G shows a representative immunoblot of α-syn and tubulin (tub, protein load control) protein expressions. Values in (**G**–**I**) are expressed as percent of vehicle-treated control (CT) cells (taken as 100) and are the means ± SEM of 3 independent experiments performed in triplicates. ***, P < 0.001; ****, P < 0.0001. α-syn protein expression (**J**, N = 8, unpaired t test) and promoter activity (**Κ**, N = 18, unpaired t test) in U87 transiently transfected with XBP1S (XBP1) or an empty vector (EV). Values are expressed as percent of EV-transfected control (EV) cells (taken as 100) and are means ± SEM of 4 (**J**) or 6 (**K**) independent experiments performed in duplicates/triplicates, respectively. **, P < 0.01; ****, P < 0.0001. **L** Upper panel represents the 5’ untranslated region of human SNCA transcript variant 1 (NM_000345) with the position ( + 1 given to the start position for translation) of three potential XBP1S binding sites referred as 3, 2 and 1 that are deleted (Δ3, Δ2, Δ1) in the lower panel. Gray boxes indicate the position of the first (ex 1) and second (ex 2) exons. The lower panel provides the quantification of the human *SNCA* promoter activity in U87 cells co-transfected with either EV or XBP1S expressing vector and wild-type (WT) or deleted (Δ3, Δ2, Δ1) *SNCA* promoter (N = 15, Kruskal-Wallis test, Dunn’s multiple comparison test). Values are expressed as percent of wild-type SNCA promoter activity (taken as 100) and are the means ± SEM of five independent experiments performed in triplicates. **, P < 0.001; ns, non-statistically significant.

In order to evaluate the potential of α-syn as a therapeutic agent in glioma-related malignancies, we examined the potential of α-syn to act in concert with TMZ, a first-choice drug used to treat GBM. Figure 4A confirms that α-syn decreases GBM proliferation, and importantly, enhances the antiproliferative effect of TMZ. Interestingly, the potentiation of α-syn antiproliferative effect is likely due to TMZ-mediated upregulation of *SNCA* transcription. Thus, TMZ increases α-syn protein (Fig. 4B) and mRNA levels (Fig. 4C). In agreement with our data in Fig. 3, TMZ also decreases CCND1 protein (Fig. 4D) and mRNA levels (Fig. 4E). TMZ induces ER stress [29] and α-syn has been linked to the UPR response [30]. Thus, we examined whether this cellular process could account for TMZ-mediated control of α-syn. We first explored the impact of TMZ in the regulation of a master transcription factor mediator of the UPR response, XBP1. Figure 4F–I shows that TMZ increased *XBP1* mRNA levels (Fig. 4F). Thus, we assessed whether pharmacological blockade of spliced and active XBP1 (XBP1S) could affect α-syn-expression. Panels 4G, H first confirm that TMZ increases α-syn protein (Fig. 4G) and *SNCA* mRNA (Fig. 4H) levels. Of importance, the specific inhibitor of XBP1 activity toyocamycin (TO) reduces α-syn protein and mRNA expressions (Fig. 4G, H see TO lanes) and fully blocks TMZ-mediated α-syn up-regulation (Fig. 4G, H, compare TMZ vs TMZ/TO lanes). Figure 4I confirms the transcriptional effect of TMZ on *XBP1* mRNA regulation (Fig. 4F) and demonstrates that TO treatment reduces *XBP1* mRNA levels (Fig. 4I, compare CT vs TO lanes), and prevents the TMZ-mediated increase in *XBP1* mRNA expression (Fig. 4I, compare TMZ vs TMZ/TO lanes). Two independent lines of evidence indicate that XBP1S likely controls *SNCA* transcription directly. First the overexpression of the XBP1S constructs triggers an increase of α-syn protein (Fig. 4J) and promoter activity (Fig. 4K) in U87 cells; second, mutational analysis approach shows that the deletion of one of the XBP1 response element located at −1006/−1003 region (see Δ3 region in Fig. 4L upper panel) but not more 3’ responsive elements (see Δ2 and Δ1 in Fig. 4L and expression of proteins Fig. S6) identified in silico [31] significantly reduces XBP1-mediated *SNCA* promoter transactivation (Fig. 4L).

Finally, to assess the possible synergistic effects of pharmacological ER stress inducers and TMZ, we have treated U87-sensitive cells with thapsigargin (TP), an irreversible inhibitor of Ca2 + -ATPase (SERCA), alone or in combination with TMZ. Data shown Fig. S7 shows that, as expected, TMZ alone triggers an increase of α-syn (Fig. S7A,B) and a decrease of cyclin D1 levels (Fig. S7C,D). The combination of TMZ and TP treatments does not amplify the impact of TMZ, suggesting that the combination of TMZ with the genetic modulation of either XBP1 or α-syn likely remains the most pertinent therapeutic track.

### α-synuclein reverses TMZ-associated resistance of GBM cells

**Fig. 5:**
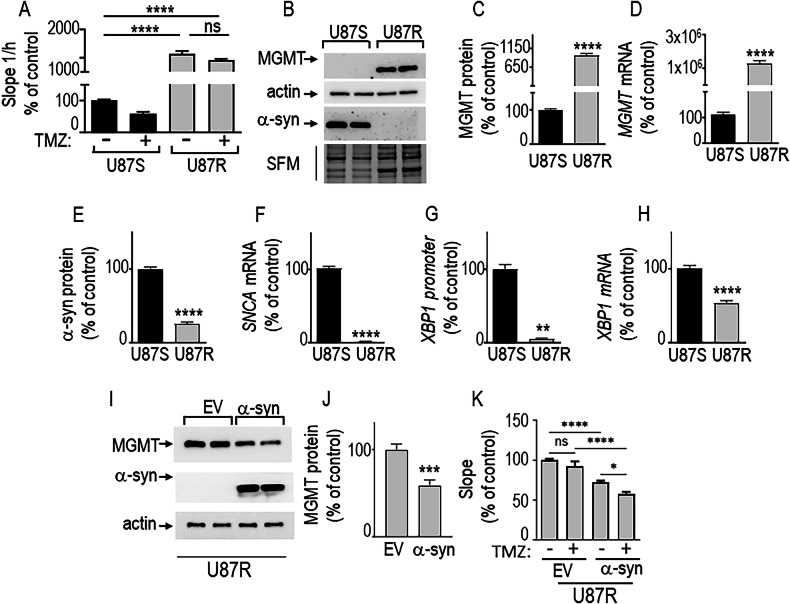
α-synuclein reverses TMZ-associated resistance of GBM cells. **A** Analysis of cellular proliferation in TMZ-sensitive parent U87 (U87S) and in TMZ-resistant U87 cells (U87R). **A** N = 8, One-way ANOVA, Dunnet’s multiple comparison test). Proliferation of U87S and U87R cells treated with vehicle or with TMZ (100 µM) was assessed as described in “Materials and Methods”. Values represent means of the slope values ± SEM of 2 independent experiments performed in quadruplicates and are expressed as percent of control vehicle-treated U87S (TMZ: U87S) taken as 100%. ****, P < 0.0001; ns, non-statistically significant. **B**–**F** Protein and mRNA expressions of MGMT (B-D) and α-syn (B,E,F) in U87S and U87R cells. Actin expression serves as a loading control and is used for normalization. Protein expressions (**C**, **E**, N = 12, unpaired t test) are expressed as percent of corresponding U87S cells (taken as 100) and represent the means ± SEM of at four independent experiments performed in triplicates. *MGMT* (**D**, N = 15, Mann–Whitney test) and *SNCA* (**F**, N = 12, unpaired t test) mRNA levels are expressed as percent of U87S cells (taken as 100) and represent the means ± SEM of at 4–5 independent experiments performed in triplicates. ****, P < 0.0001. mXBP1 promoter activity (**G**) and XBP1S mRNA expression (**H**) in U87S and U87R cells. *mXbp1s* promoter activity (**G**, N = 6, unpaired t test) is expressed as percent of U87S cells (taken as 100) and represent the means ± SEM of at least 2 independent experiments performed in triplicates. **, P < 0.01. *Xbp1s* (**H**, N = 12, unpaired t test) mRNA levels are expressed as percent of U87S cells (taken as 100) and represent the means ± SEM of at least 3 to 4 independent experiments performed in triplicates. ****, P < 0.0001. Impact of α-syn complementation on MGMT protein expression (**I**, **J**) and proliferation (**K**) in U87R cells. **I** α-syn, MGMT and actin (protein charge load control) protein levels. **J** MGMT expression (N = 9, Mann–Whitney test) is expressed as percent of EV-transfected cells (taken as 100), and represent the means ± SEM of at least 3 independent determinations in triplicates. ***, P < 0.001. **K** Proliferation rate (slope) of control or α-syn-expressing U87R cells treated (+) or not (–) with TMZ (100 µM, 48 h, N = 8, One-way ANOVA, Sidak’s multiple comparison test). Values are expressed as percent of vehicle-treated EV-U87R cells (taken as 100) and represent the means ± SEM of 2 independent experiments performed in quadruplicate. *, P < 0.05; ****, P < 0.0001.

As α-syn mimics and even potentiates TMZ-induced protection against proliferation (see Fig. 4), we examined whether α-syn could overcome TMZ-associated resistance developed after long-term treatments. We took advantage of control TMZ-sensitive (U87S) and TMZ-resistant (U87R) GBM models developed by one of us [32]. At first, we examined their TMZ-response in terms of proliferation, Fig. 5A shows that U87R proliferate faster than U87S cells in basal conditions (compare U87R vs U87S in Fig. 5A. As expected, although, U87S proliferation was significantly reduced by TMZ, this agent did not modify U87R proliferation (Fig. 5A, compare U87S + vs U87R +). Second, we have evaluated the expression of MGMT, an enzyme, the increased expression of which has been associated with TMZ resistance. As expected, U87R cells display drastically higher MGMT protein (Fig. 5B, C) and mRNA levels (Fig. 5D). Interestingly, besides this well-known TMZ-resistance marker, α-syn protein (Fig. 5E) and mRNA (Fig. 5F) levels were also reduced in U87R cells in agreement with the reduction of XBP1 promoter activity (Fig. 5G) and mRNA levels (Fig. 5H) Of note, U87R rescue of α-syn deficiency decreased MGMT levels (Fig. 5I, J) and decreased proliferation rates in basal and TMZ conditions (Fig. 5K). Overall, this data indicates that α-syn has the potential to reverse TMZ-induced resistance in GBM cells.

### Impact of α-synuclein in tumor progression in vivo

**Fig. 6:**
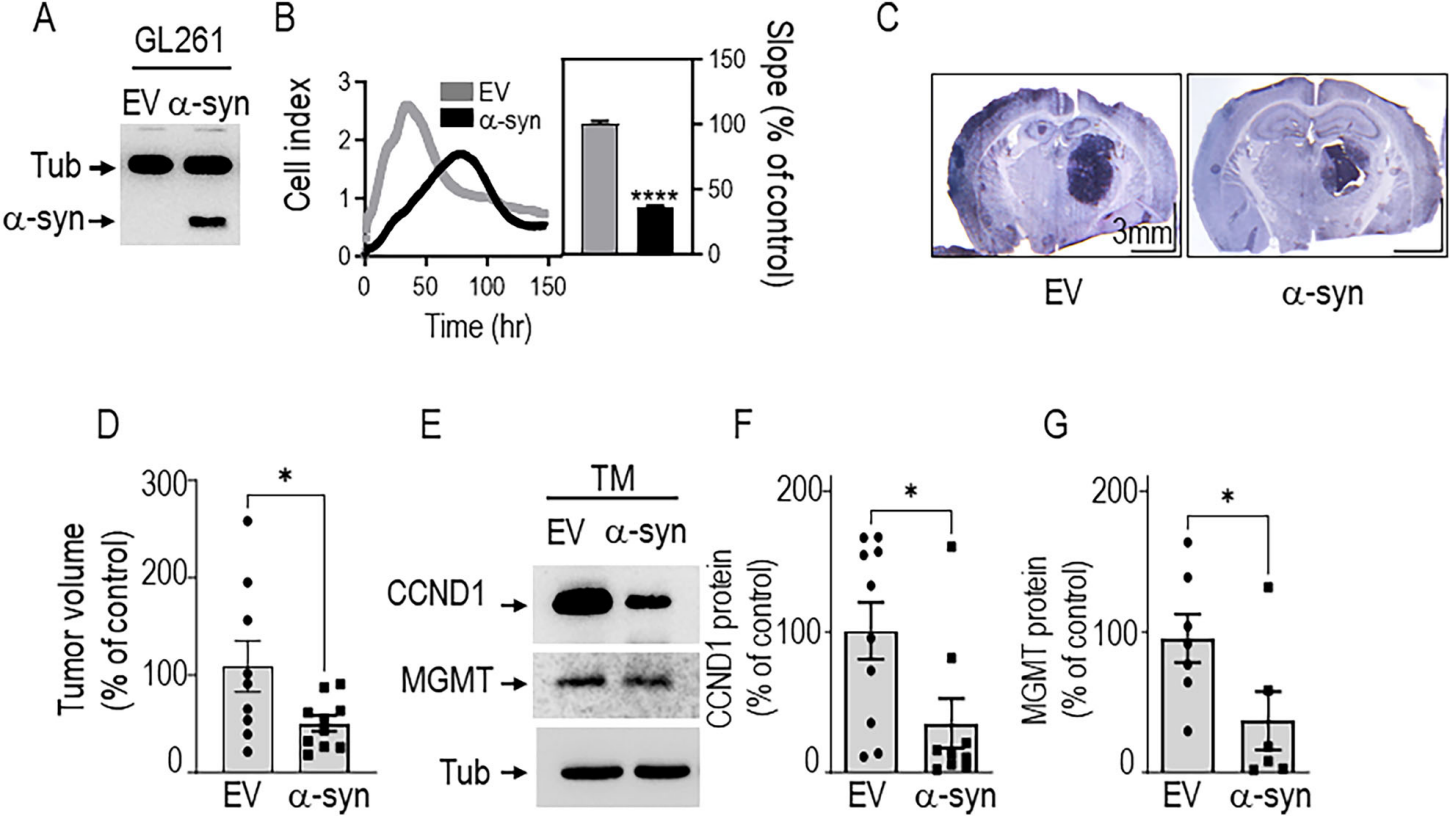
α-Syn induces tumor suppression and reduces cyclin D1 and MGMT expressions, in vivo. **A**, **B** Characterization of GL261 murine cells transduced with an empty Fuw (EV) or α-syn expressing vector. **A** Representative Western blot illustrating the stable overexpression of α-syn in transduced GL-261 murine cells. **B** Left panel shows the representative proliferation curves of GL261 cells overexpressing empty vector (EV) (gray curve) or α-syn (black curve). Value in the right panel corresponding to slopes quantification analysis (N = 8, unpaired t test) are expressed as percent of that obtained for EV-transduced GL261 cells (taken as 100) and represent the means ± SEM of 2 independent determinations in quadruplicates. ****, P < 0.0001. **C**, **D** Analysis of tumor volume in mice brain injected with GL261 GBM cells transduced with the empty Fuw (EV) or α-syn-expressing viruses. **C** Illustration of Hematoxylin and Eosin staining of 30 µm brain sections (Scale bar = 3 mm). **D** Quantification of tumor volume in brains injected with GL261 cells transduced with Fuw (EV) or α-syn viruses (N = 9, unpaired t test). *, P < 0.05. Cyclin D1 (**E**, **F**) and MGMT (**E**, **G**) expressions in mice brains of Fuw (EV) or α-syn-injected GL261 cells. Representative immunoblots (**E**) and quantitative analysis (**F**, **G**, (N = 9, Mann–Whitney test)) of cyclin D1, MGMT and tubulin (tub, protein load control) in the core (TM) of tumors samples. values are expressed as percent of EV-injected mice brain expressions (taken as 100) and represent the means ± SEM of 2–3 independent experiments. *, P < 0.05; ns, non-statistically significant.

Finally, to assess the therapeutic impact of α-synuclein treatment in vivo, we allografted a murine model of GBM (GL261) overexpressing either a control (EV) or an α-syn vector in wild-type immunocompetent mice (Fig. 6A). First, we confirmed that these cells show decreased proliferation in α-syn overexpressing conditions (Fig. 6B). Importantly, the injection of GL261 α-syn overexpressing cells reduces tumors size (Fig. 6C representative image and Fig. 6D quantification analysis of the tumoral volume), indicating that α-syn indeed slowed down tumor progression in vivo. Of importance, this was accompanied by a significant reduction of CCND1 (Fig. 6E, F) and of MGMT (Fig. 6E, G) expressions in the core of mice brain tumors (TM) thereby corroborating, in an integrated context, data obtained in vitro.

## Discussion

α-syn belongs to a family of highly homologous small proteins mainly expressed in the brain, the physiological function of which remains largely unknown. Interestingly, a number of theoretical grounds support the possible link between α-syn and cancer. Thus, multiple studies indicated that the protein levels of several members of the synuclein family are affected in various types of cancers. This was particularly well documented for γ-synuclein that is highly expressed in various types of cancer from central and peripheral origins, particularly at late stages of high grade gliomas [33]. Reports on the alteration of α-syn expression in cancers are less numerous but it has been reported that α-syn is abnormally expressed in cancers including ovarian, breast, colorectal and skin tumors [11–13].

Our study shows that the levels of α-syn are reduced in oligodendrogliomas and glioblastoma. This reduction occurs at early stages and appears grade-dependent (Fig. 1F). Our data is supported by the work of Fung et al. who analyzed synucleins-like immunoreactivity by immunohistochemistry in astrocytomas and oligodendrogliomas [34]. Their qualitative study revealed α-syn-like expression in all types of tumors without examining the correlation of α-syn levels to tumor grades. Our in-depth quantitative study filled this important gap and indicates that α-syn could be envisioned as an early marker of glioma setting and progression as it has been proposed for γ-synuclein, which is now recognized as a cancer progression biomarker in breast cancer and is generally associated with poor prognosis [35]. Importantly, the analysis of α-syn expression in multiple databases corroborates our experimental data and indicates that α-syn is more expressed in low grade gliomas and samples harboring mutations in the *IDH* gene and a 1p/19q codeletion, than in GBM. The common feature comes from the SNCA expression in all IDH-_mut_ gliomas, expressing early stages oligodendrocyte lineage genes and are stalled in oligodendrocyte differentiation [36] and specifically in the OPC-like subpopulation of GBM. The oligodendrocyte signature evidenced here strongly suggests that higher α-syn expression is linked to a better prognosis in gliomas [37]. Further, studies will be necessary to definitively establish its prognostic role in gliomas and other cancers since its regulation could vary according to the tumor type. For example, α-syn is highly expressed in melanoma cells [38].

We have previously shown that p53 and α-syn could regulate each other in a neuronal context [7, 17]. It was therefore tempting to speculate that the decrease of α-syn levels in human biopsy samples (Fig. 1E, F) could be accounted for by the loss of p53 transcriptional function. In line with this statement, it is important to emphasize that p53 expression/activity is commonly affected in gliomas [21]. Thus, the study of p53 targets may lead to the development of alternative therapeutic strategies that would bypass the fact that p53 reactivation therapeutic strategies could trigger numerous deleterious side-effects linked to its large spectrum of physiological targets.

We show here that defective p53 activity is associated with a decrease in α-syn protein, promoter activity and mRNA levels in multiple GBM cellular models. We show that p53 activates α-syn transcription in a glial tumor context ex-vivo and in vivo and that activity-deficient p53 mutant failed to display this regulation in mice brain. It is worth noting that p53 inactivation by mutations is a key mechanism of glioma genesis [39, 40]. Overall, this strongly suggests that the down-regulation of α-syn observed in human biopsies is indeed linked to p53 transcriptional loss of function.

This study unravels that the physiological interplay between α-syn and p53 is not restricted to neurons and its disruption in glial cells may be at the origin of brain tumors. Corroborating this hypothesis, we demonstrate that α-syn harbors tumor suppressor properties and can decrease tumor progression. Thus, as demonstrated in Fig. 3, α-syn overexpression in U87 GBM cells decreases cell proliferation and increases caspase-3 activity, two key parameters involved in the process of cell transformation. This α-syn-associated tumor suppressor phenotype and decreased expression in human glioma biopsies flags-up α-syn as a novel early glioma biomarker but also opens a new brain tumors therapeutic track.

It is interesting to note that other PD-causative gene products have been recognized as important glioma-associated tumor suppressors. Thus, parkin, a PD causative gene also capable of functionally interacting with p53 [8, 17] has been identified as a tumor suppressor in GBM [10, 20, 41].

We were able to decrypt the mechanisms by which α-syn could modulates cell proliferation in GBM. α-syn reduced cyclin D1 protein and mRNA levels as well as its promoter transactivation (Fig. 3). Cyclin D1 is a key modulator of cell cycle dynamics involved in cancer [42]. It is noticeable that α-syn-mediated transcriptional regulation of cyclin D1 is compatible with the reported nuclear localization of α-syn [43] and its ability to directly [44] or indirectly control gene expression [45, 46]. Importantly, it should be noted that several works have demonstrated that the levels of cyclin D1 are increased in human glioma biopsies [47, 48] that could well be a consequence of the reduction of α-syn protein levels. Interestingly, α-syn downregulates cyclin D1 levels in GBM cells in contrast to neuronal cells suggesting a cell type regulation [27]. Additionally, we demonstrate that the control of cell proliferation by α-syn is dependent of cyclin D1 since cyclin D1-depletion in U87 cells abolishes α-syn-mediated reduction of cell proliferation, a phenotype restored in cells in which cyclin D1 expression was restored. Considering that cyclin D1 is transcriptionally regulated by TP53 [26], it was important to establish if α-syn acted downstream or upstream to p53 in the control cell proliferation and cyclin D1. We show that overexpression of α-syn in a GBM cell model that harbor transcriptionally inactive p53 (8MG, Fig. S4) can decrease cell proliferation via a down regulation of cyclin D1 indicating that α-syn functions downstream to p53 in the control of cell proliferation. This information is quite important if one considers that a therapeutic strategy aimed at increasing of α-syn expression and thereby restore its tumor suppressor function would potentially be successful whatever the mutational/functional status of p53.

TMZ is a first-choice therapeutic drug used in clinics, but its main drawback is that GBM may become resistant TMZ-associated therapy explaining the frequent relapse of this uncurable type of cancer. Thus, we examined whether targeting α-syn could interfere with TMZ and whether α-syn could overcome TMZ-associated resistance (Fig. 4). We show first that α-syn potentiates TMZ-associated antiproliferative effect. This was emphasized by the ability of TMZ to increase α-syn protein and mRNA expression. As expected from our demonstration of an α-syn-mediated reduction of cyclin D1, TMZ also reduced cyclin D1 protein and mRNA levels.

Interestingly, several studies evidence the contribution of ER-stress activation and an enhanced unfolded protein response (UPR) in gliomagenesis [49]. The latter process could account for either an adaptative survival response, under mild stress, or a pro-apoptotic response, when the capacity of the ER to deal with stress is exceeded [50]. XBP1 is a transcription factor and master regulator of the UPR. The involvement of XBP1S-mediated ER-stress response in both TMZ-induced response and control of α-syn was demonstrated by five lines of independent evidences. First, toyocamycin (TO), a selective blocker of IRE1α-induced XBP1 mRNA cleavage lowers α-syn protein and mRNA levels; second, TO abolishes TMZ-induced control of α-syn; Third, TMZ triggers an increase of XBP1 mRNA levels, a phenotype abolished by TO; Fourth, XBP1 increases α-syn and mRNA levels; Fifth, we identified three XBP1-responsive elements on the α-syn 5’UTR region and the deletion of one of them fully prevents XBP1-mediated control of α-syn. The fact that both α-syn accumulation and TMZ have been linked to ER stress activation strongly suggests that the potentiation of TMZ effect by α-syn may be linked to a bypass of the adaptative UPR response. Corroborating an impact of α-syn in the reversal of a pharmacological resistance to TMZ, we show that α-syn overexpression in U87 resistant cells trigger a down-regulation of MGMT, a key prognostic marker of GBM response to TMZ, and that it restores TMZ ability to reduce GBM cells proliferation.

Finally, we validated the therapeutic potential of α-syn in vivo. Thus, we showed that the injection of GBM cells overexpressing α-syn drastically lowers the tumor volume and reduces cyclin D1 levels in vivo model. Of importance, in vivo administration of α-syn also lowers MGMT, thus corroborating the protective role of α-syn against tumor development.

Overall, our work identifies α-syn as a tumor suppressor in glioblastomas and demonstrates its therapeutic impact in vitro and in vivo. This finding may open new alternatives for GBM treatment and the bypassing of TMZ resistance. Finally, the potential of α-syn as a regulator of glioma proliferation in a p53-deficient context allows opening up the opportunity for new treatments whatever the functional p53 status and glial cellular origin of the tumor.

## Supplementary information

Supplementary Methods_Figure legends and references

Figure S1

Figure S2

Figure S3

Figure S4

Figure S5

Figure S6

Figure S7

Uncropped WB

## Data Availability

All data are available in the main text or the supplementary materials. Human and mouse glioblastoma transfer is restricted due to materials transfer agreements (MTAs). The data may be available upon reasonable request.
