# Supplementary material for: α-synuclein expression in glioblastoma restores tumor suppressor function and rescues temozolomide drug resistance
Source: Cell Death Dis. 2025 Mar 19;16(1):188. doi: 10.1038/s41419-025-07509-z (PMC11923286; doi:10.1038/s41419-025-07509-z)
Supplement: Supplementary file 1 — Supplementary Methods_Figure legends and references [file 41419_2025_7509_MOESM1_ESM.pdf]

SUPPLEMENTARY METHODS

In silico analysis* of *SNCA expression in gliomas from available public databases.

SNCA gene expression, molecular and clinical data on normal brain tissue (n=5) and primary lower grade gliomas IDH_{mut}-codel (n=168) or IDH_{mut}-non codel (n=246) and GBM IDH_{wt} (n=231) patients were downloaded from The Cancer Genome Atlas (TCGA) Research Network https://www.cancer.gov/tcga). *In silico* analysis of *SNCA* mRNA expression in the different human specimens was done from the gene expression array platform Affymetrix HT Human Genome U133a of GBM patients with known survival data, tumor recurrence information, from TCGA (1). Proneural, Neural, Classical and Mesenchymal subtypes were collected from UCSC Xena database (2). For glioma patient survival analysis, primary non-recurrent tumors cases, both IDH-$_{mut}$ and IDH-$_{wt}$, of patients who did not receive treatment prior to resection and survived more than 30 days after resection were selected. Patient survival was defined as the time between histological diagnosis and death. Differences between Kaplan-Meier survival curves were assessed using the log-rank (Mantel-Cox) test. We also used the accessible single-cell database arising from the molecular information of GBM cell heterogeneity from the publication by Darmanis et al. (3). A total of 3,589 cells in a cohort of four patients with two separate GBM tissue samples: one originating from the tumor core and another from the peritumoral space (cortex) immediately adjacent to the tumor core, were analyzed by scRNAseq. mRNA levels were represented as fragments per kilobase of transcript per million mapped reads (FPKM) corresponding to a normalization of a RNAseq expression level read counts. Data were downloaded from the Gephart lab portal (http://www.gbmseq.org/) and illustrated in **Figure S2B** Single cell analysis of another cohort of 28 adult and pediatric high-grade gliomas from the publication by Neftel et al (4) was also used. T-stochastic neighborhood embedding plots (tSNE), lineage plots and dot-plots were done with PRISM10 Graphpad from data imported from the Broad Institute Cell portal interface (https://singlecell.broadinstitute.org/single_cell/study/SCP393/single-cell-rna-seq-of-adult-and-pediatric-glioblastoma). The processed logTPM expression matrix, hierarchy data and metadata of the cohort of 20 adult GBM patients was here used. In addition, *SNCA* gene

expression obtained from 4 GBM patients was analyzed from the IVY Glioblastoma Atlas Project (IVY gap) and the database available from the IVY gap website (https://glioblastoma.alleninstitute.org/static/download.html). Gene expression was obtained by RNA sequencing extracted from different GBM areas (cellular tumor, perinecrotic zone, pseudopalisading cells, hyperplastic blood vessels, microvascular proliferation, infiltrating tumor and leading edge) isolated by laser micro-dissection (Technical White Paper: overview – 2015, glioblastoma.alleninstitute.org). For comparison with normal tissue, Genome-Tissue Expression (GTEx) database accessible from the GTEx portal (https://gtexportal.org/home/) was also used to illustrate the distribution of *SNCA* mRNA expression levels in brain and other organs (**Figure S2D**). This *in silico* analysis is schematized in **Fig. S1**.

Cellular models and pharmacological treatments.

Human glioblastoma (U87, U87-temozolomide (TMZ)-resistant and control cells, 8MG), neuroblastoma SH-SY5Y cells and mouse glioma cell lines (LN215, GL261), 3T3 cell line, were cultured in Dulbecco's modified Eagle's medium (DMEM) supplemented with 10% fetal calf serum, penicillin (100 U/ml) and streptomycin (50 µg/ml) and incubated at 37°C in a 5% CO_2 atmosphere.

U87, 8MG, GL261, LN215, 3T3 cells (MC14 cyclin D1-/- infected either with MSCV-GFP control vector, or with MSCV-cyclin D1-expressing vector) and U87-TMZ-control and resistant cells were provided by three of us (5, 6). The human cell lines used in this study were authenticated by genetic profiling of short tandem repeat (STR) loci according to manufacturer’s conditions (GenePrintR 10 System, Promega) with the ABI PRISM 3100 Genetic Analyzer. Each cell line was routinely tested at the beginning and each 6 months of continuous utilization as recommended by National Institutes of Health.

In a subset of experiments, U87 cells were treated with of Temozolomide (TMZ, 100 µM, 48 hours SIGMA, #T2577) and/or of Toyocamycin (TO, 1 µM, 24 hours, SIGMA, #T3580) or with the appropriate vehicles. In Fig. S7, TMZ-sensitive cells were treated with 100 µM of TMZ (or DMSO) for 24 hours and 7 hours later with 1 µM of Thapsigargin (TP) or DMSO for 17 hours. TMZ, TP, TMZ/TP or DMSO (CT) treated cells were all harvested at the same time and processed as described in the Material and Method part for Western-blot experiments.

Plasmid constructs and virus production.

Sub-cloning of human wild-type p53 (flag-tagged) or its R175H mutant in the pcDNA3.1 (+) vector has been extensively described (7). 5'UTR of "human *SNCA* promoter" was cloned between the Mlu I and Bgl II sites of pGL3 basic vector (E175, Promega). Briefly we used a preparation of human genomic DNA (extracted from SH-SY5Y cells) (QIAamp DNA mini kit, 51304, Qiagen) with the primers "5'UTR" described in Table 1 of the supplementary Methods. The amplicon of 1043 nucleotides generated by PCR corresponds to the 5'untranslated region above the start codon of α-syn (isoform NM_000345). This human *SNCA* regulatory region was used as a template to generate, by site-directed mutagenesis, the selective deletion of each of the three putative XBP1S binding sites. Primers used to delete XBP1S site 3 (hSNCAprΔ-1005/-1003), site 2 (hSNCAprΔ-692/-690F) and site 1 (hSNCAprΔ-236/-234) are described in Table 1 of the supplementary Methods. The vector pA3-1745 CD1 Luc containing human CCND1 promoter region (portion -1745 to +134) was generated by one of us and described in (8). The FUW lentiviral empty vector EV, (gift from David Baltimore, Addgene plasmid # 14882) (9) was used to insert between HpaI and AscI sites, human α-syn coding sequence (NM_000345) and mouse XBP1S coding sequence (NM_013842). To overexpress α-syn and XBP1S, lentiviruses were produced and used to transduce various cell types as previously described (10). Viral titers, determined by p24 ELISA, were around 5 x 10^7 transduction units per ml *(TU/ml)* for all virus preparations. Cells were transduced (for 4 days) with 10 µl of control, α-syn or XBP1S lentiviruses.

The primers used in this study are depicted below (Table 1).

Primers used for cloning or mutagenesis		
**ADN**	**FORWARD PRIMER**	**REVERSE PRIMER**
5'UTR human SNCA	5'-CGACGCGTGGAAATCCTGGAGAACGCCG-3'	5'-GAAGATCTGGCTAATGAATTCCTTTACACCACACTGG-3'
XBP1S site 3	5'-GGGAGACGAATGGTGGCACCGGGAGGGG-3'	5'-CCCCTCCCGGTGCCACCATTCGTCTCCC-3'
XBP1S site 2	5'-CAAATAATGAAATGGAAAAGGAGGCCAAGTCAAC-3'	5'-GTTGACTTGGCCTCCTTTTCCATTTCATTATTTG-3'
XBP1S site 1	5'-GAGGATTCATCCTAATCCGTTAGGTGGCTAGACTTT-3'	5'-AAAGTCTAGCCACCTAACGGATTAGGATGAATCCTC-3'

Patient's biopsies cohort.

The cohort used in this study has been extensively characterized concerning the mutational status of p53 and extensively described in (7). In brief, the cohort gathered by the tumor bank (obtained from the Centre de Recherche de l'Institut du Cerveau et de la Moelle Epinière, Groupe Hospitalier Pitié-

Salpêtrière UMRS 975, Paris, France) consists of 47 patients who underwent a craniotomy for tumor resection during the 2004-2012 period. Only patients satisfying the following inclusion criteria were kept for our study: minimal age of onset of 18 years-old, diagnosis of gliomas according to the World Health Organization (WHO) criteria and available clinical data. This cohort includes 17 oligodendrogliomas (10 of grade II and 7 of grade III), 11 grade IV (glioblastomas) and 20 control (non-related epilepsy surgery-derived samples) samples. These samples were snap-frozen and stored at -80°C immediately after surgical resection. High molecular weights DNA were isolated from both tumor and peripheral blood using a standard phenol-chloroform procedure. A written informed consent from all patients and a validation by a local ethical committee were obtained for this study allowing molecular, genetic and translational research studies on cancer tissue samples.

SNCA genomic BAC ARRAY-CGH and TP53 mutation analysis.

A full-coverage of genomic bacterial artificial chromosome (BAC) array (CGH) with an average resolution of 1 Mb, previously described (11), was used for DNA copy number analysis. The procedures for DNA extraction, hybridization and washing have been described previously (12). Arrays were scanned using a 4000B scan (Axon, Union city, USA). Image analyses were performed with Genepix 5.1 software (Axon, Union city, USA) and ratios of Cy5/Cy3 signals were determined. The ratio of Cy5/Cy3 of the BAC including or contiguous to the target gene investigated was attributed to the gene of interest. A ratio above at least 3,0 was fixed as the threshold to conclude that a gene amplification genuinely occurred. Data were normalized with MicroArray NORmalization: MANOR and BAC status (gained, lost, amplified, or normal) was determined using GLAD, and the final results were visualized with VAMP software. *TP53* coding exons of all tumors and paired-blood plasmas DNAs were analyzed by Universal Tailed Amplicon Sequencing approach (454 Sequencing Technology, Roche) and confirmed by direct genomic sequencing of both DNA strands by the Sanger method as extensively described in (7).

Protein analysis by Western-blot.

Actin, α-syn, cyclin D1 (CCND1), MGMT, p53, tubulin and XBP1S expressions were analyzed in cell lines, mice brain homogenates and human biopsies. Briefly, 50 µg of total proteins from lysed samples were loaded on 12-16% SDS-PAGE gels (containing 10% v/v of 2,2,2- Trichloroethanol (TCE; Sigma

T54801)). For loading normalization, proteins in the gel were photoactivated allowing Stain-Free imaging (Bio-Rad's protocol) and semi-dry transferred by means of the precut blotting transfer pack (Bio-Rad) and the Trans-Blot® Turbo™ Transfer System (Bio-Rad's protocol for 2 mini gels of 1.5 mm). Transferred proteins were blocked for one hour in 1X TBS (containing 5% w/v nonfat dry milk) and immunobloted over-night at 4°C with the following primary antibodies (anti-actin (clone AC-74, A5316, Sigma), anti-α-syn (#2642, Cell Signaling), anti-CCND1 (clone EPR2241, # 04-1151, Millipore), anti-flag (clone M2, F3165, Sigma), anti-MGMT (Santa Cruz Biotechnology, sc-56432), anti-p53 (CM1, provided by Dr J.C. Bourdon), anti-tubulin (Sigma-Aldrich, T5168) and anti-XBP1 (BioLegend, # 658802). Immunological complexes were revealed with either adequate anti-rabbit or anti-mouse IgG-coupled peroxidase antibodies (Jackson ImmunoResearch) by the electrochemiluminescence detection method (Roche Diagnostics S.A.S). Chemiluminescence was recorded using a luminescence image analyzer LAS-4000 (Raytest, Fuji) or a ChemiDoc™ Imaging System (Bio-Rad) and quantifications were performed with the FUJI Film Multi Gauge image analyzer or the Image lab 6.0.1 software. For some experiments instead of using actin or tubulin as a loading control, we used stain-free imaging. All full gels are provided as supplemental material.

Luciferase-based reporter assays.

PG13 (containing 13 copies of p53 DNA-binding site (13)), the 1745 bp *hCCND1* promoter, the 1043 bp *hSNCA* promoter (wild type or mutated at one of the three XBP1 putative binding sites) or the m*XBP1* promoter (region of mouse *Xbp1* −689 to +37 bp, a generous gift from Dr Ling QI) coupled to the luciferase reporter gene were used to measure either *TP53* transcriptional activity or *hCCND1*, *hSNCA* and m*XBP1* promoter activities. In a subset of experiments cells were co-transfected by means of lipofectamine 2000 (Invitrogen) according to the manufacturer's instructions (Invitrogen) with 1 μg of the above gene reporter constructs and 0,5-0,8 μg of vectors expressing either WT p53, R175H p53, α syn or XBP1S. Transfection of 0,2-0,5 of β-galactosidase cDNA was used to normalize transfection efficiencies. In experiments described in Figure 5G cells were co-transfected with 1μg of the mXBP1 promoter and 1μg of β-galactosidase cDNA to normalize transfection efficiencies Twenty-four hours after transfection,

cells were lysed and β-galactosidase and luciferase activities were performed according to manufacturer's instructions by means of E 2000 and E 1500 kits (Promega), respectively. Protein concentrations were evaluated using the Bradford method.

RNA extraction, reverse transcription and real-time PCR analysis.

RNA from cells and mice brains were extracted and treated with DNAse using RNeasy or RNeasy Plus Universal Mini kits respectively following manufacturer's instructions (Qiagen). Two µg of total RNA were reverse transcribed (GoScript Reverse Transcriptase, Promega) using Oligo-dT priming then, samples were subjected to real-time PCR, and analyzed by means of a Rotor-Gene 6000 apparatus and software (Qiagen), using the SYBR Green detection protocol. Gene-specific primers were designed with the Universal ProbeLibrary Assay Design Center software (Roche Applied Science). mRNA expression levels were normalized for RNA concentrations by mRNA expression levels of mouse or human housekeeping genes TBP or GAPDH. All primers used in this work are described in supplementary TABLE 2 below.

Primers for QPCR		
**GENE**	**FORWARD PRIMER**	**REVERSE PRIMER**
h CCND1	5′-TCACACGCTTCCTCTCCAG-3′	5′-CAGGGCTTCGATCTGCTC-3′
m Ccnd1	5′-TTTCTTTCCAGAGTCATCAAGTGT-3′	5′-TGACTCCAGAAGGGCTTCAA-3′
h MGMT	5′-GTGATTTCTTACCAGCAATTAGCA-3′	5′-ACTCTGTGGCACGGGATG-3′
m Mgmt	5′-CTGGAAGCCTATTTCCGTGA-3′	5′-TCCATAACACCTGTCTGGTGAA-3′
h XBP1S	5′-AGCTTTTACGAGAGAAAACTCA-3′	5′-GCCTGCACCTGCTGCG-3′
h SNCA	5′-GCCTTTCCACCCTCGTGAG-3′	5′-ACTGTCGTCGAATGGCCAC-3′
m snca	5′-TGGCAGTGAGGCTTATGAAA-3′	5′-GCTTCAGGCTCATAGTCTTGG-3′
h GAPDH	5'-TGGGCTACACTGAGCACCAG-3'	5'-CAGCGTCAAAGGTGGAGGAG-3'
m gapdh	5′-AAGAGGGATGCTGCCCTTAC-3′	5′-CCATTTTGTCTACGGGACGA-3′
h TBP	5′-ATTGGTGTTCTGAATAGGCTGTG-3′	5′-GAACATCATGGATCAGAACAACAG-3′
m Tbp	5′-GGGGAGCTGTGATGTGAAGT-3′	5′-CCAGGAAATAATTCTGGCTCAT-3′
h Flag-p53	5'-CAGCCACCTGAAGTCCAAAAAG-3'	5'-CTTGTCGTCATCGTCTTTGTAGTC-3'

Allografts and in vivo transfection.

C57BL6/JRj wild-type adult (two months) mice (Janvier-Labs) were housed in alternating cycles (12 hours of light/ 12 hours of darkness) and had access to water and conventional food *ad libitum*. For allograft experiments, ten non randomized male mice (number established with Gpower program) per group were anesthetized intraperitoneally with a mixture of Ketamine (150 mg/kg) and Xylazine (10

mg/kg) after a subcutaneous injection of lidocaine at the microsurgery site, then, stereotaxically injected at the striatum (right hemisphere (ML: 1.6 mm, AP: 1 mm, DV: 3.2 mm from bregma) with 10^5 murine GBM cells transduced with viruses containing either empty FUW vector or human α-syn cDNA (GL261-EV or GL261-αSyn). An anti-inflammatory agent (Carprofen, 10 mg/kg) was provided in drinking water following tumor implantation. Animals were daily followed and sacrificed 14 days after injection.

For *in vivo* transfection experiences, eight animals of each group (control, wild-type, and mutated R175H p53) were anesthetized intraperitoneally as above and injected into the striatum, using appropriate coordinates (1 mm anterior to the bregma, 2 mm lateral, 3 mm deep from the skull surface) by means of thin walled borosilicate glass capillary tubes fitted to the Microinjection Unit. Endotoxin free DNA constructs were delivered to mice brain by means of *in vivo*-JetPEI protocol. In brief, DNA (1.5 µg)/PEI complexes (4 µl/mice hemisphere) prepared according to manufacturer's conditions to achieve an N/P (number of nitrogen residues of *in vivo*-jetPEI™ per phosphate in the nucleic acid) ratio of eight (Polyplus transfection), were injected at a flow rate of 0,5 µl/min. Animals were sacrificed two days after injection and tumor volume and protein analysis performed in non-blinded conditions.

Tumor volume analysis.

Mice were anesthetized intraperitoneally as above and sacrificed by intracardiac perfusion. Organs were fixed with 4% PFA, brains recovered and frozen in liquid nitrogen. Coronal sections (30 µm) of tumors were performed with a cryostat, recovered on SuperFrostPlus slides (VWR International, Radnor, PA, USA) and a Hematoxylin -eosin (H&E) staining was done. Pictures were taken with an Olympus MXV10 macroscope (Shijuku, Tokyo, Japan), objective 2X and processed with Fiji software (ImageJ 1.53t, National Institute of Health, USA; Java 1.8.0_172 64bit, (14). The software was calibrated with a reference image then for each section of a tumor, an area value (a) (mm^2) is evaluated. A volume (v) (mm^3) per section is calculated (v = a*0.030). To obtain the total tumor volume "V" in mm^3, the formula is: V = v*n.

Statistical analysis.

Statistical analyses were performed with GraphPad Prism software (version 9.00 for Windows, San Diego, California USA). The Gaussian distribution of values was evaluated by the normality test (D'Agostino-Pearse omnibus Normality test). Two groups of variables that have passed the normality test were analyzed by unpaired Student's t-test. Two groups that have not passed the normality test were analyzed by the Mann-Whitney test. More than two groups of variables that have passed the normality test were analyzed by One-way ANOVA while, if they have not passed the normality test, we used a Kruskal-Wallis test (post-tests provided in results section). All tests are two-sided; the mean was defined as the center value and error bars correspond to SEM. The number of samples and replication of experiments are provided in figure legends. Significant differences were settled as: *p<0.05, **p<0.01, ***p<0.001, ****p<0.0001 and ns, non-significant.

SUPPLEMENTARY FIGURE LEGENDS

Fig. S1: Flow chart of data processing from publicly available RNA-seq datasets from gliomas specimens. Association of SNCA expression level with glioma patient survival from TCGA clinical information.

Fig. S2 α-synuclein levels are reduced in human brain glioma.

(**A**) Kaplan-Meier survival curve illustrating the prognostic significance of chromosomal arms 1p and 19q codeletion in IDH_{mut} glioma patients (IDH_{mut}-codel). The overall survival of patients with IDH_{mut}-codel glioma was significantly higher than IDH_{mut}-non codel glioma patients (P=0.0122). Blue line, low expression; red line, high expression; *n* indicates the number of samples in each low or high expression groups.

(**B**) 2D-tSNE representation of all single cells (n=3589 from 4 GBM samples) included in the study of Darmanis et al. (2017). Clusters are identified as indicated distinct cell classes. Histograms of SNCA expression (log2 Counts Per Million) in neurons, oligodendrocytes, OPCs and myeloid cells.

(**C**) IVY-gap database analysis of the SNCA differential expression over GBM microenvironment represented by the color code of the indicated structural zones, histologically evaluated, processed with laser-microdissection, and analyzed by RNA-seq.

(**D**) Analysis of the Genome-Tissue Expression (GTEx) database. Note that *SNCA* is more expressed in tissue from the brain compared to other tissues and organs.

Fig. S3 *In vivo* transfection of p53 or its mutant R175H (175) in mice brain.

Control (EV-FLAG) wild-type (p53) or mutated p53-FLAG (175) in pcDNA3 vector (DNA 1.5 µg/PEI complexes) were injected into the striatum. Eight animals were sacrificed two days after injection. For each mouse, one hemisphere was processed for protein analysis and one for mRNA analysis. Bars on the histogram show the means ± SEM of the relative quantification of the FLAG mRNA expression in eight hemispheres. * = $P < 0.05$.

Fig. S4 Characterization of 3T3 cyclin D1 KO and complemented cells.

(**A**) Representative Western blot of 3T3 cells depleted in cyclin D1 ($D1^-$) stably expressing a viral control vector ($D1^-$/CT) or cyclin D1 (CCND1, $D1^-$/CP).

(**B**) Effect of endogenous cyclin D1 depletion (CT, in grey) and rescue (CP, in black) on proliferation of 3T3 cells as described in Methods (N=18, impaired t-test). Bars correspond to quantification analyses of the slopes of the curves and correspond to the means ± SEM of six independent experiments performed in triplicates.

(**C,D**) α-syn expressions in 3T3 cells either depleted (**C**) in CCND1 ($D1^-$/CT) or complemented (**D**) for CCND1 after transduction of empty (EV) or α-syn-bearing viral vectors. Tubulin expression is provided as a control of protein charge load in (A, C, D).

Fig. S5 Impact of p53 mutation to the α-syn-mediated control of GBM proliferation.

(**A**) Effect of viral infection of 8MG cells with an empty (EV, gray curve and bar) or α-syn expressing vector (α-syn, black curve and bar) on cellular proliferation assessed by impedance-based label free approach as described in Materials and Methods. Quantification analyses of the slopes of the curves

are provided. Bars correspond to the means ± SEM of 3 independent experiments performed in triplicates.

(**B-E**) Evaluation of α-syn impact on caspase 3 activity (B, **N**=9, unpaired t-test), cyclin D1 (CCND1) protein (C, **N**=9, unpaired t-test), promoter (D, **N**=6 Mann Whitney test) and mRNA expressions (E, **N**=6 Mann Whitney test) in the 8MG models described above. Data are expressed as percent of EV control cells (taken as 100%) and are the means ± SEM of 3 independent experiments performed in triplicates.

Fig. S6 Transfection of XBP1 in U87 cells.

(A) Representative immunoblot showing XBP1S protein expression in co transfection experiments with the wild-type (WT), Δ1, Δ2, Δ3 hSNCA 5'UTR portion described in Fig.4L. The stain free membrane (SFM) is used as a protein charge loading control.

Fig. S7 Impact of ER stress to TMZ mediated α-syn and CCND1 regulations.

(**A,B**) α-syn and cyclin D1 (C,D) expressions in U87 TMZ-sensitive cells were treated with 100 μM of TMZ (or DMSO) for 24 hours and 7 hours later with 1 μM of Thapsigargin (TP) or DMSO for 17 hours. TMZ, TP, TMZ/TP or DMSO (CT) treated cells were all harvested at the same time and processed as described in the Material and Method part for Western-blot experiments.

(A-C) Representative immunoblots and quantitative analysis (B, D, (N=9, One-way ANOVA) of α-syn and cyclin D1 protein levels. Values are expressed as percent of DMSO (CT) treated cells (taken as 100) and represent the means ± SEM of 3 independent experiments. *, P<0.01; ***, P<0.001; ****, P<0.0001 and ns, non-statistically significant. Stain free membranes (SFM) are shown to demonstrated equal protein load.

SUPPLEMENTARY REFERENCES

1. Verhaak RG, Hoadley KA, Purdom E, Wang V, Qi Y, Wilkerson MD, et al. Integrated genomic analysis identifies clinically relevant subtypes of glioblastoma characterized by abnormalities in PDGFRA, IDH1, EGFR, and NF1. Cancer Cell. 2010;17(1):98-110.

2. Goldman MJ, Craft B, Hastie M, Repecka K, McDade F, Kamath A, et al. Visualizing and interpreting cancer genomics data via the Xena platform. Nat Biotechnol. 2020;38(6):675-8.

3. Darmanis S, Sloan SA, Croote D, Mignardi M, Chernikova S, Samghababi P, et al. Single-Cell RNA-Seq Analysis of Infiltrating Neoplastic Cells at the Migrating Front of Human Glioblastoma. Cell Rep. 2017;21(5):1399-410.

4. Neftel C, Laffy J, Filbin MG, Hara T, Shore ME, Rahme GJ, et al. An Integrative Model of Cellular States, Plasticity, and Genetics for Glioblastoma. Cell. 2019;178(4):835-49 e21.

5. Kohsaka S, Wang L, Yachi K, Mahabir R, Narita T, Itoh T, et al. STAT3 inhibition overcomes temozolomide resistance in glioblastoma by downregulating MGMT expression. Mol Cancer Ther. 2012;11(6):1289-99.

6. Casimiro MC, Di Sante G, Crosariol M, Loro E, Dampier W, Ertel A, et al. Kinase-independent role of cyclin D1 in chromosomal instability and mammary tumorigenesis. Oncotarget. 2015;6(11):8525-38.

7. Viotti J, Duplan E, Caillava C, Condat J, Goiran T, Giordano C, et al. Glioma tumor grade correlates with parkin depletion in mutant p53-linked tumors and results from loss of function of p53 transcriptional activity. Oncogene. 2014;33(14):1764-75.

8. Albanese C, Johnson J, Watanabe G, Eklund N, Vu D, Arnold A, et al. Transforming p21ras mutants and c-Ets-2 activate the cyclin D1 promoter through distinguishable regions. J Biol Chem. 1995;270(40):23589-97.

9. Lois C, Hong EJ, Pease S, Brown EJ, Baltimore D. Germline transmission and tissue-specific expression of transgenes delivered by lentiviral vectors. Science. 2002;295(5556):868-72.

10. Cisse M, Halabisky B, Harris J, Devidze N, Dubal DB, Sun B, et al. Reversing EphB2 depletion rescues cognitive functions in Alzheimer model. Nature. 2011;469(7328):47-52.

11. Idbaih A, Marie Y, Pierron G, Brennetot C, Hoang-Xuan K, Kujas M, et al. Two types of chromosome 1p losses with opposite significance in gliomas. Ann Neurol. 2005;58(3):483-7.

12. Idbaih A, Marie Y, Lucchesi C, Pierron G, Manie E, Raynal V, et al. BAC array CGH distinguishes mutually exclusive alterations that define clinicogenetic subtypes of gliomas. Int J Cancer. 2008;122(8):1778-86.

13. El-Deiry W, Kern S, Pietenpol J, Kinzler K, Vogelstein B. Definition of a consensus binding site for p53. Nat Gen. 1992;1:45-9.

14. Schindelin J, Arganda-Carreras I, Frise E, Kaynig V, Longair M, Pietzsch T, et al. Fiji: an open-source platform for biological-image analysis. Nat Methods. 2012;9(7):676-82.
