# Supplementary figures and images for: α-synuclein expression in glioblastoma restores tumor suppressor function and rescues temozolomide drug resistance
Source: Cell Death Dis. 2025 Mar 19;16(1):188. doi: 10.1038/s41419-025-07509-z (PMC11923286; doi:10.1038/s41419-025-07509-z)

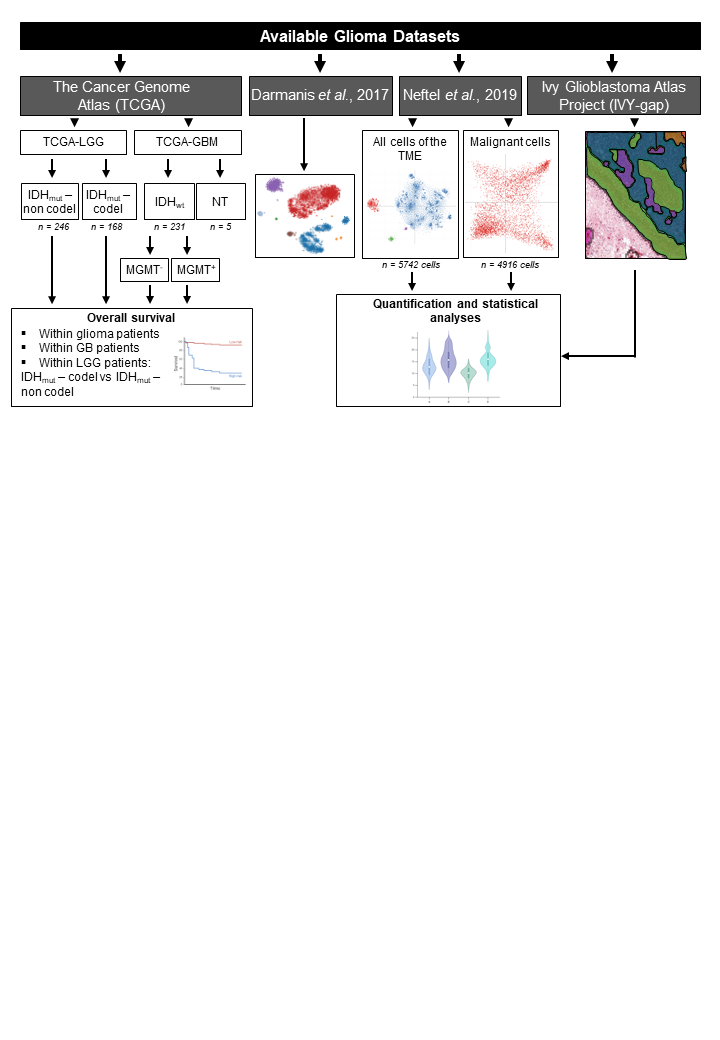

Supplement: Supplementary file 2 — Figure S1 [file 41419_2025_7509_MOESM2_ESM.tif]

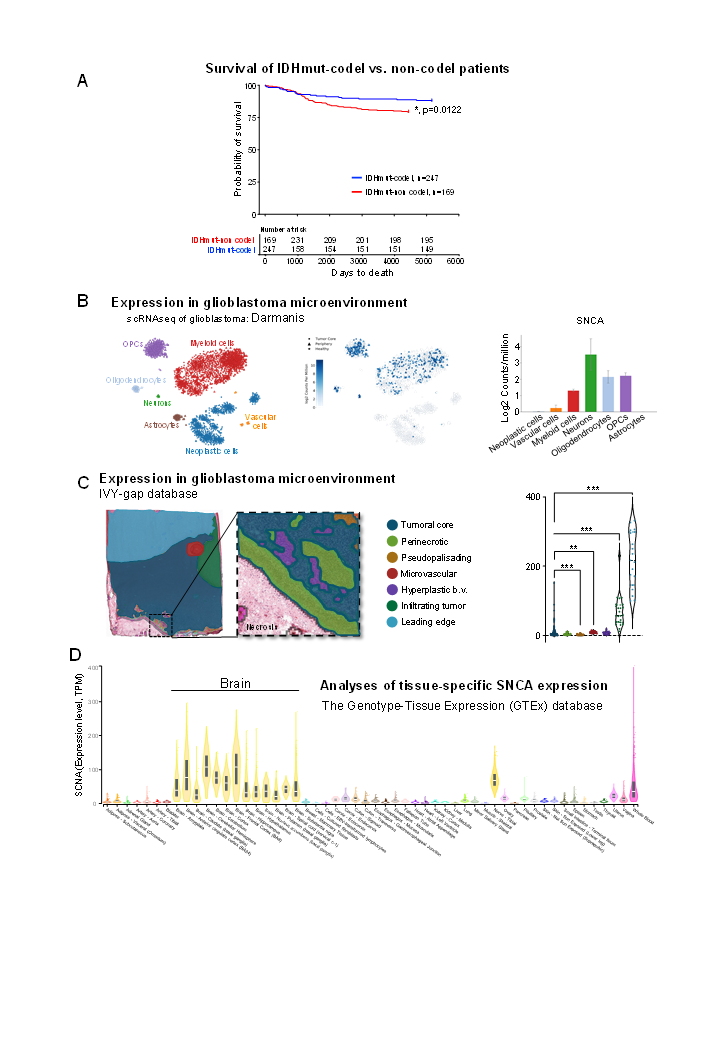

Supplement: Supplementary file 3 — Figure S2 [file 41419_2025_7509_MOESM3_ESM.tif]

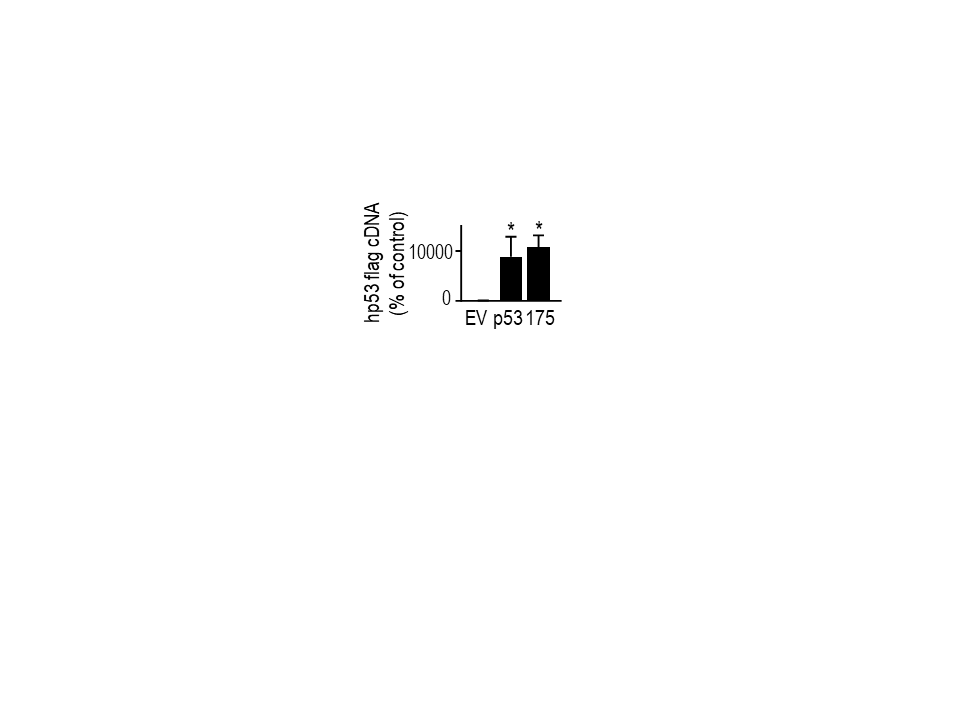

Supplement: Supplementary file 4 — Figure S3 [file 41419_2025_7509_MOESM4_ESM.tif]

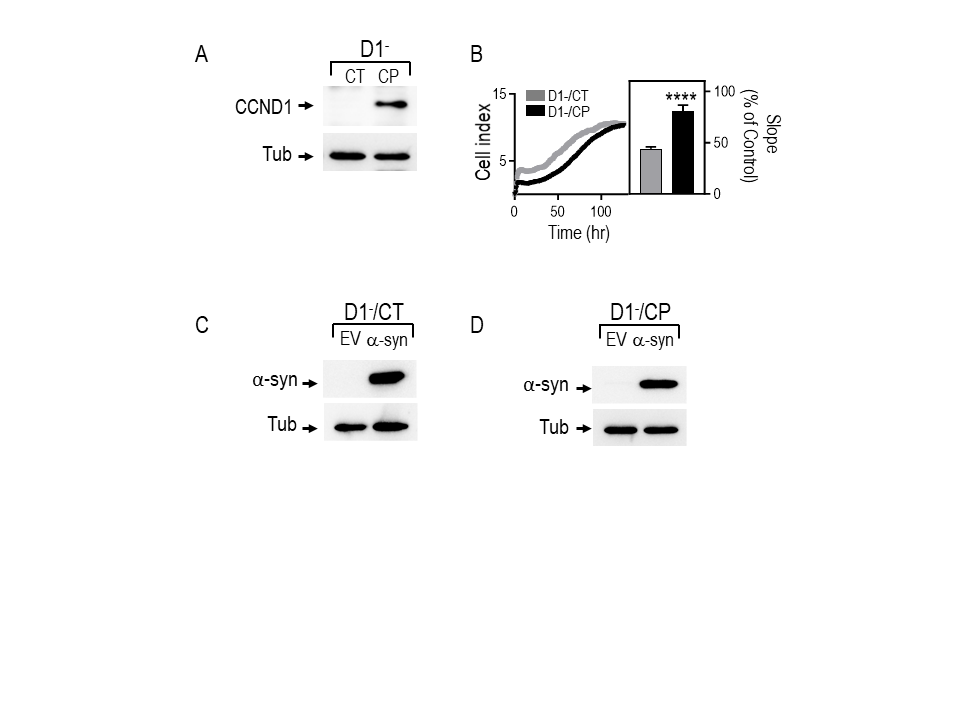

Supplement: Supplementary file 5 — Figure S4 [file 41419_2025_7509_MOESM5_ESM.tif]

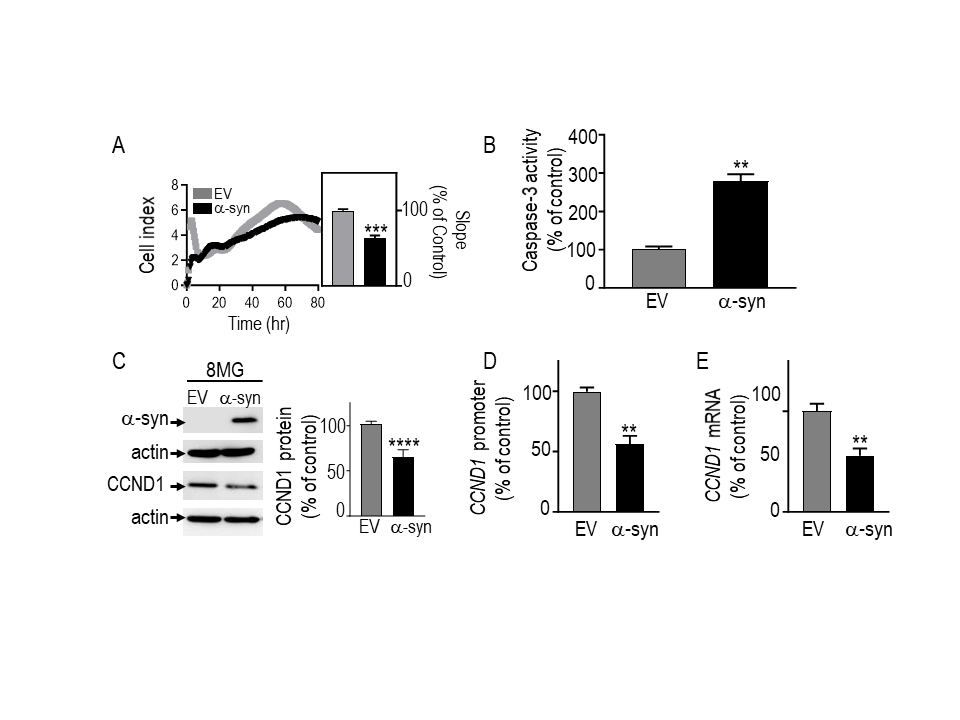

Supplement: Supplementary file 6 — Figure S5 [file 41419_2025_7509_MOESM6_ESM.tif]

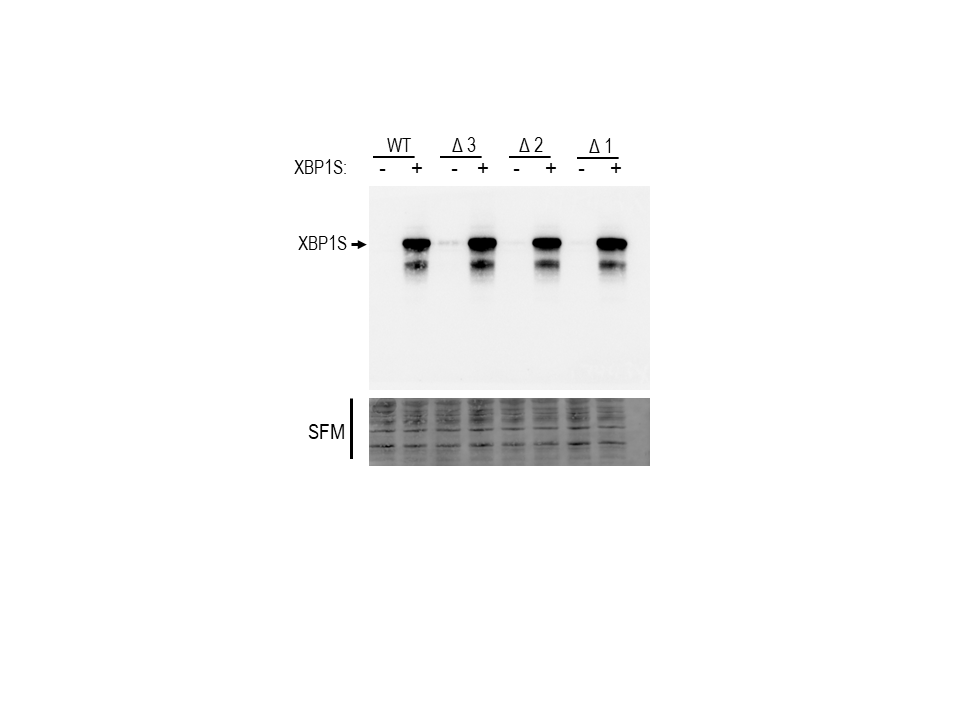

Supplement: Supplementary file 7 — Figure S6 [file 41419_2025_7509_MOESM7_ESM.tif]

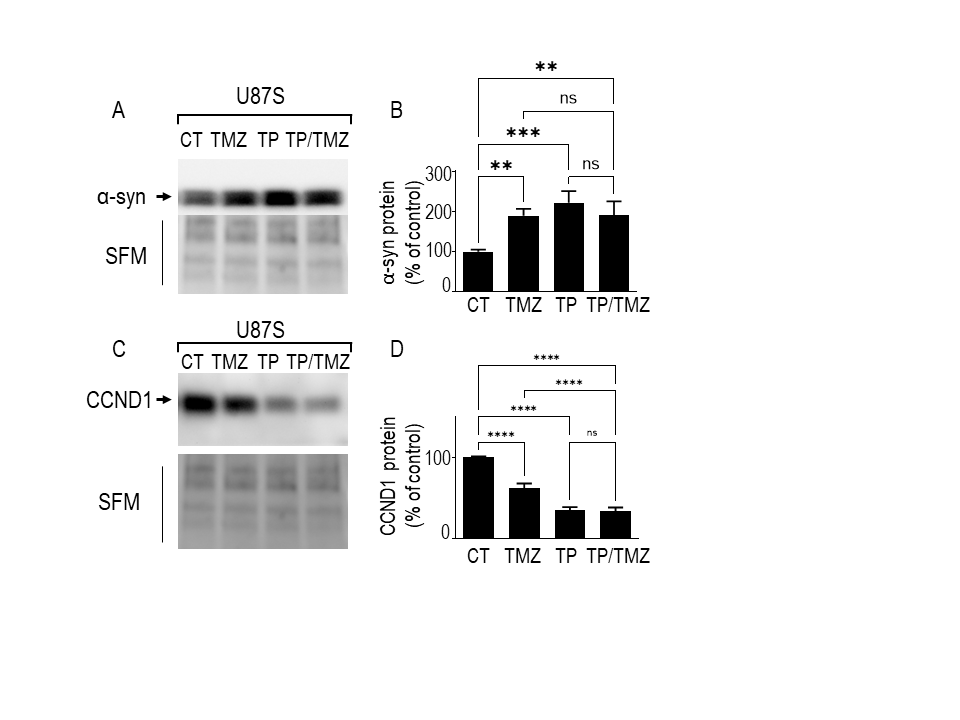

Supplement: Supplementary file 8 — Figure S7 [file 41419_2025_7509_MOESM8_ESM.tif]
