# Supplementary material for: α-synuclein expression in glioblastoma restores tumor suppressor function and rescues temozolomide drug resistance
Source: Cell Death Dis. 2025 Mar 19;16(1):188. doi: 10.1038/s41419-025-07509-z (PMC11923286; doi:10.1038/s41419-025-07509-z)
Supplement: Supplementary file 9 — Uncropped WB [file 41419_2025_7509_MOESM9_ESM.pdf]

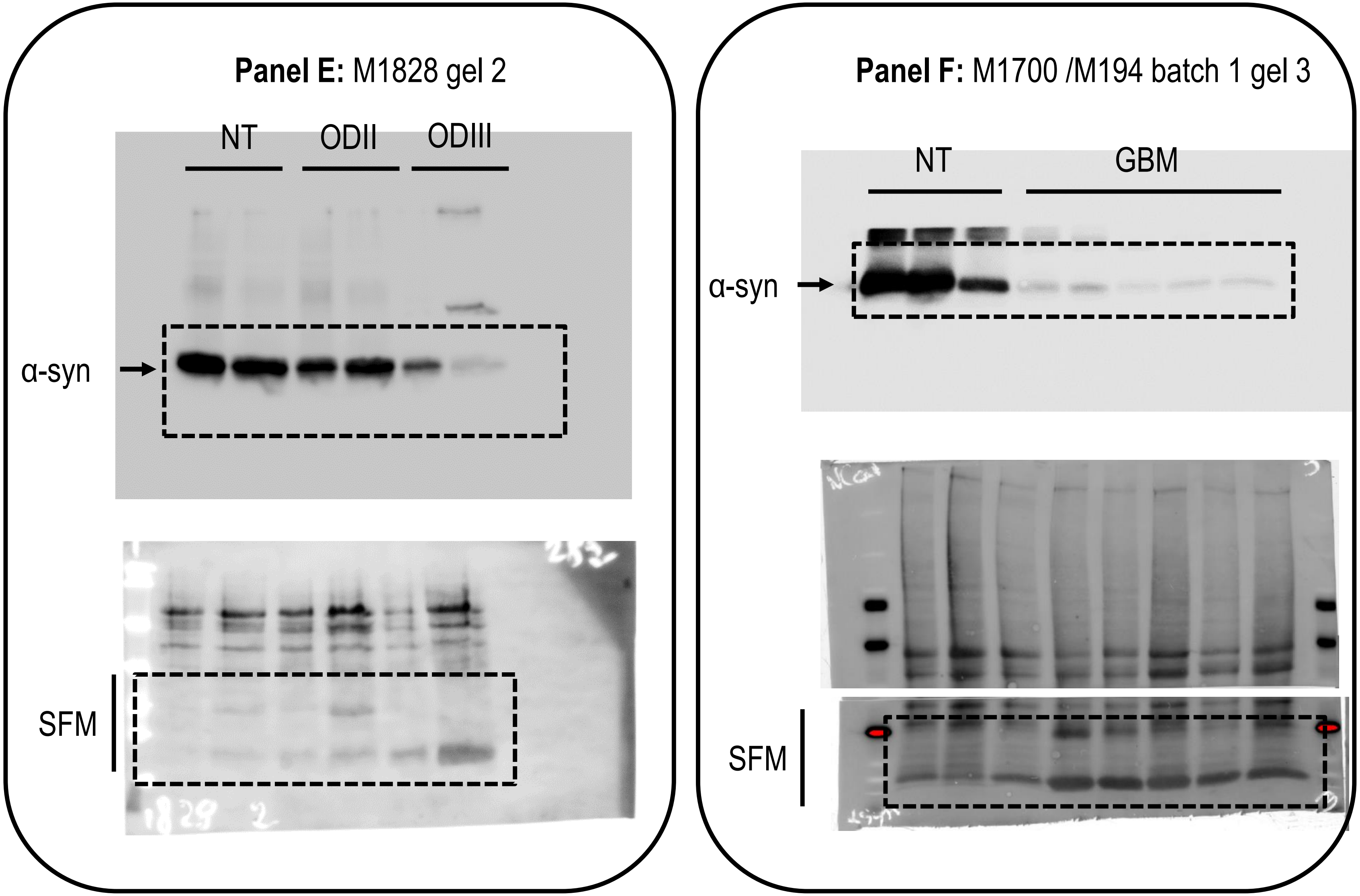

Gels for figure 1

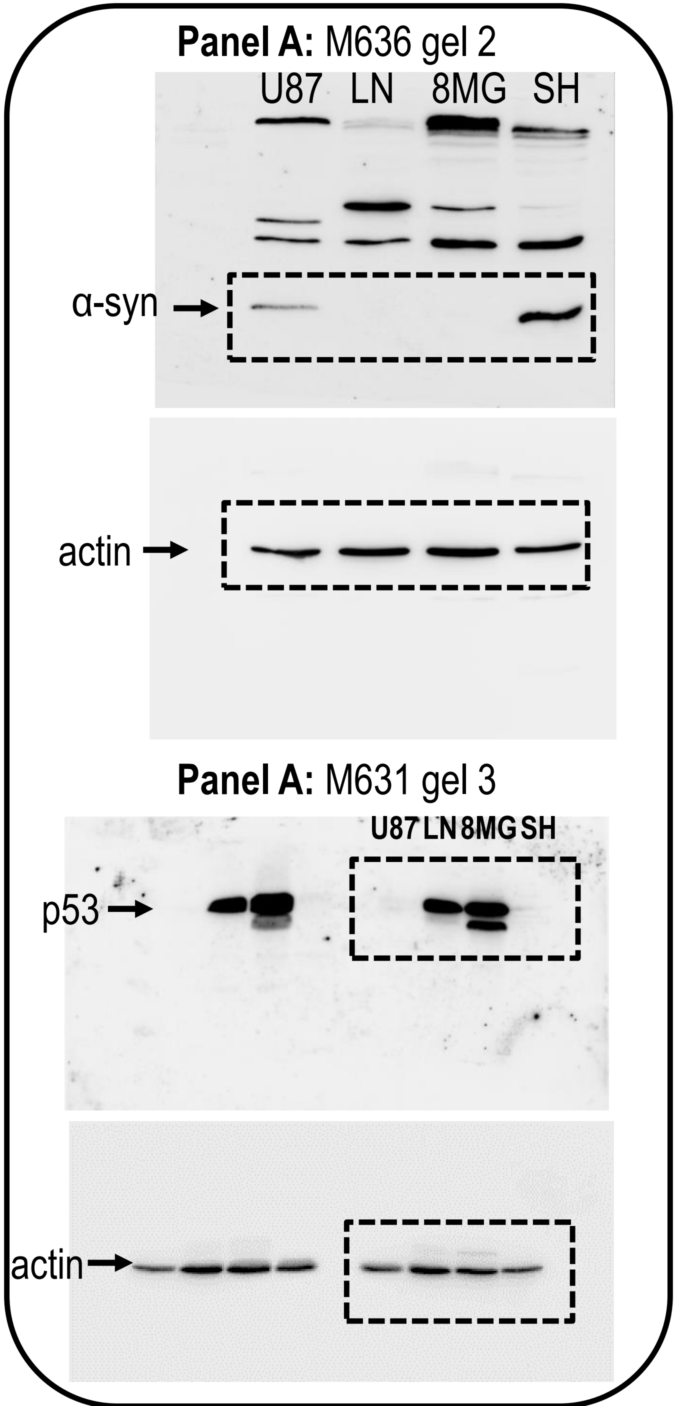

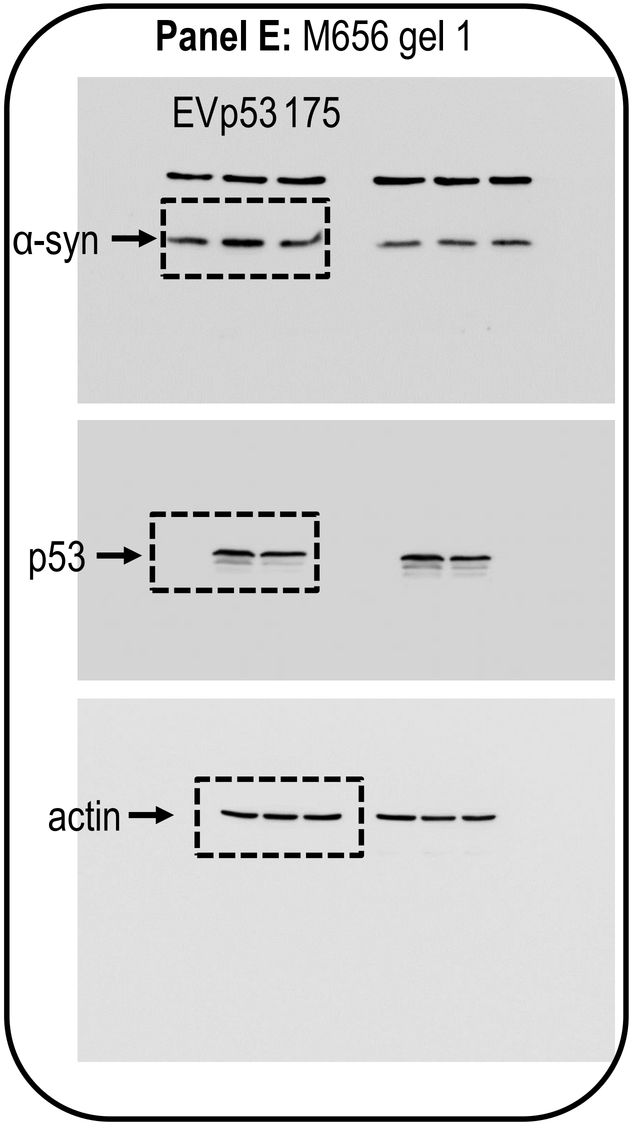

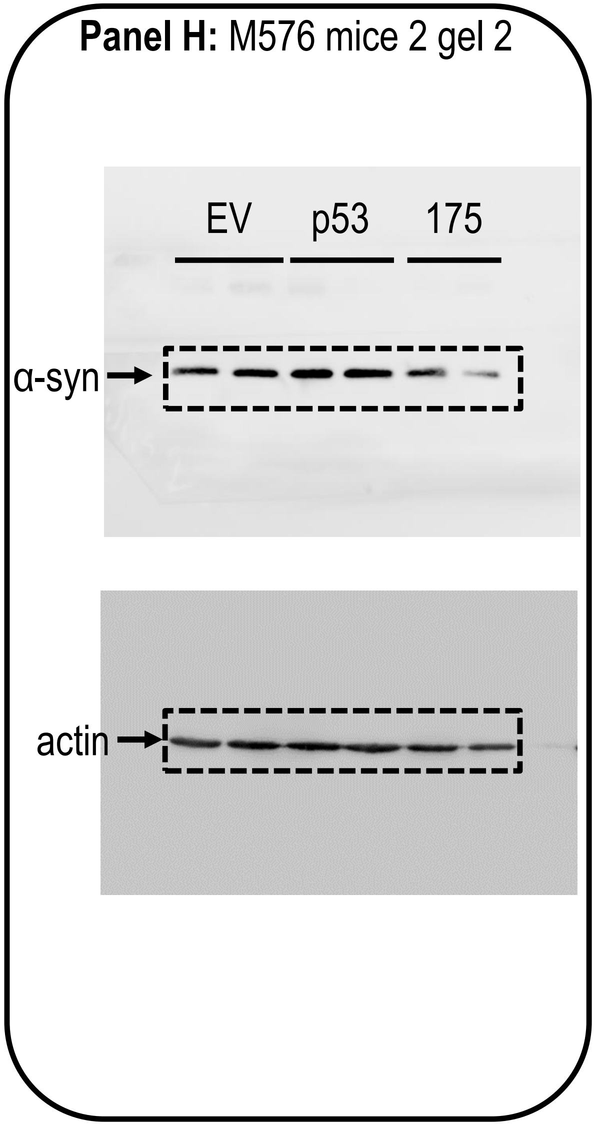

Gels for figure 2

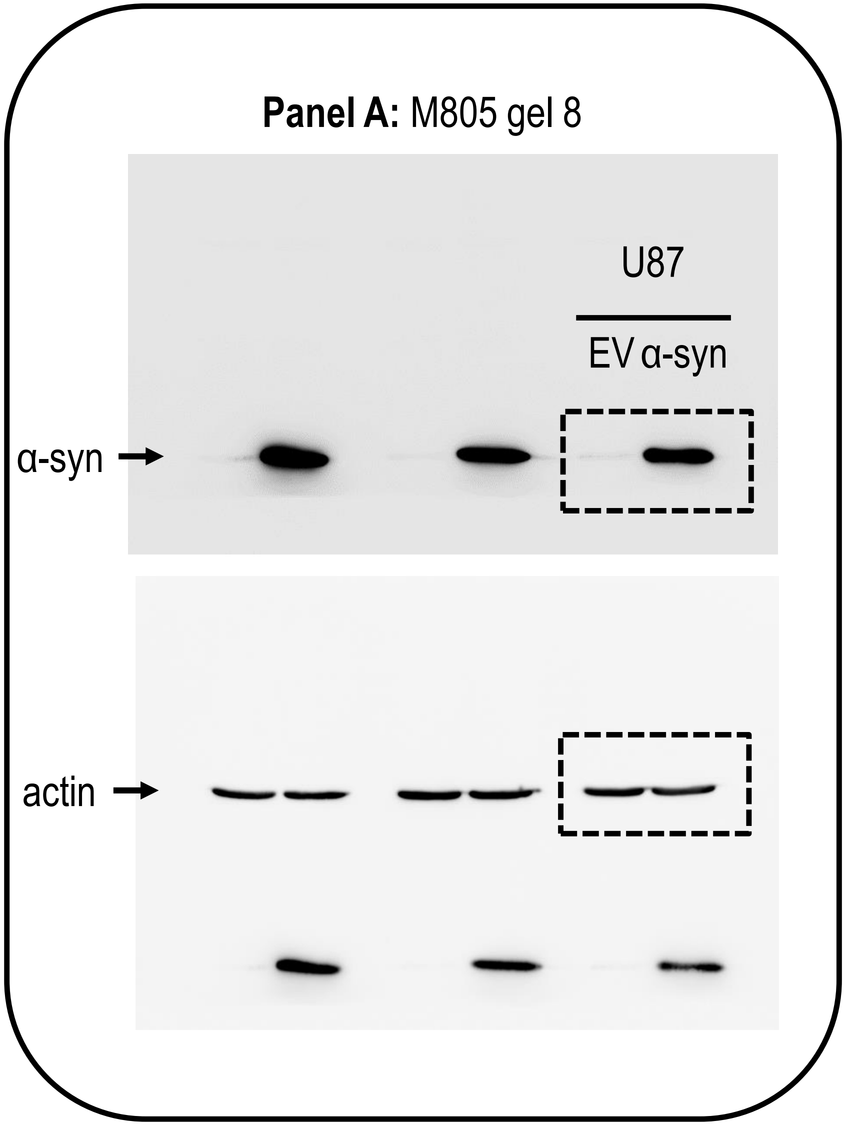

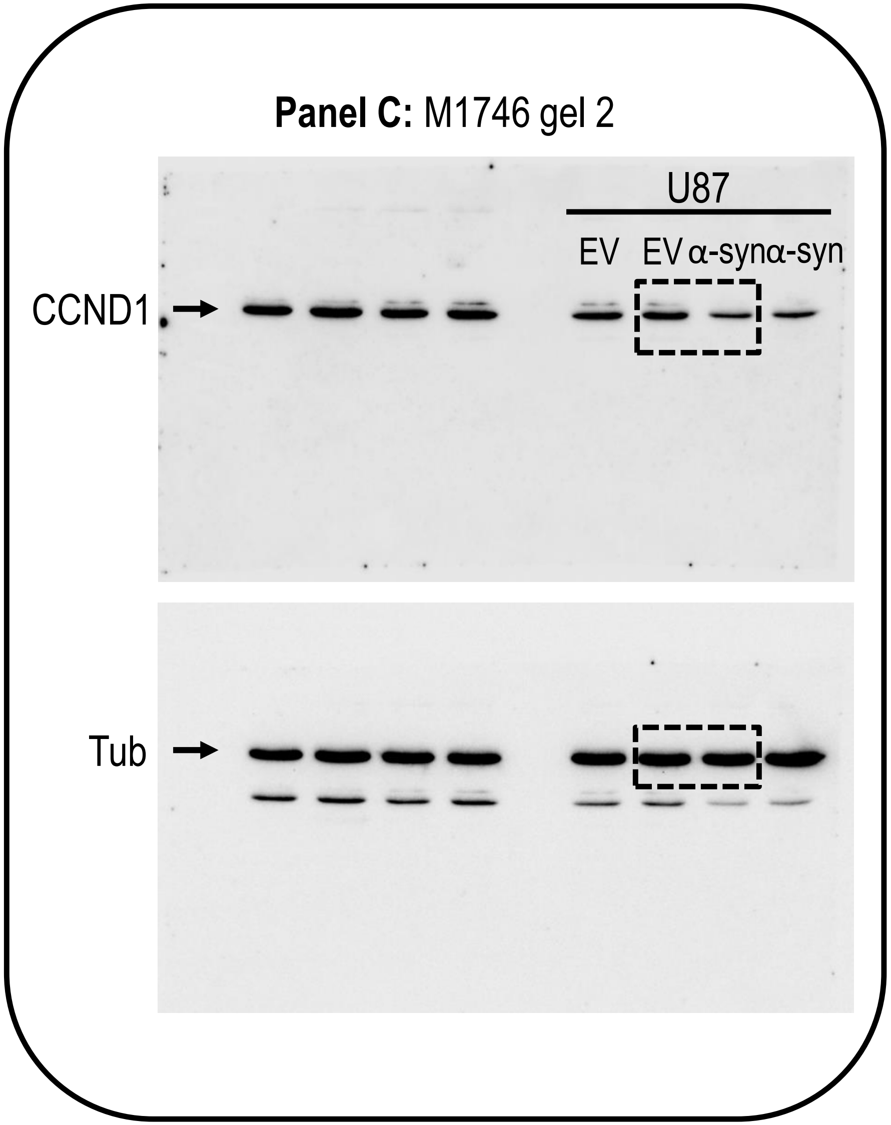

Gels for figure 3

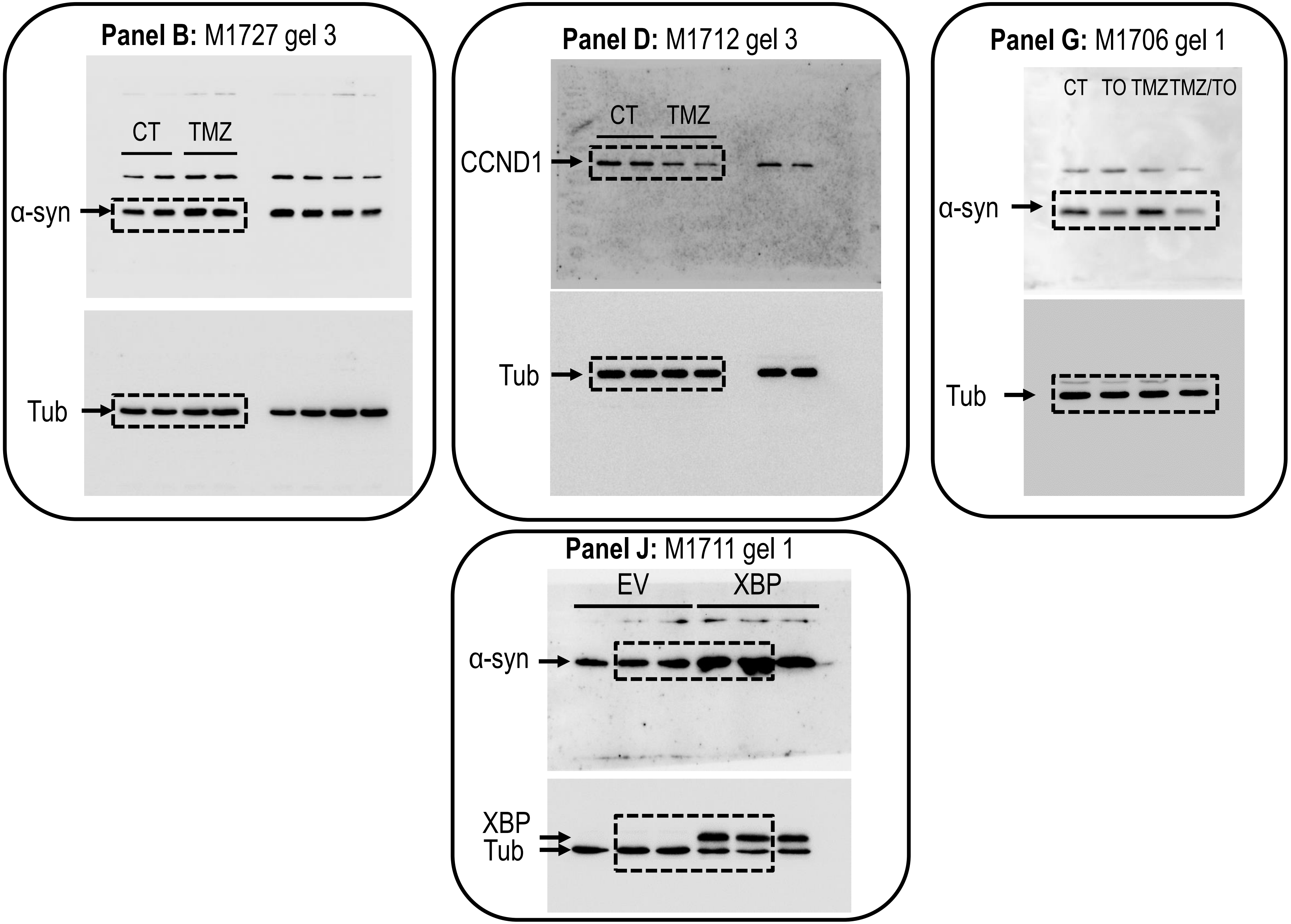

Gels for figure 4

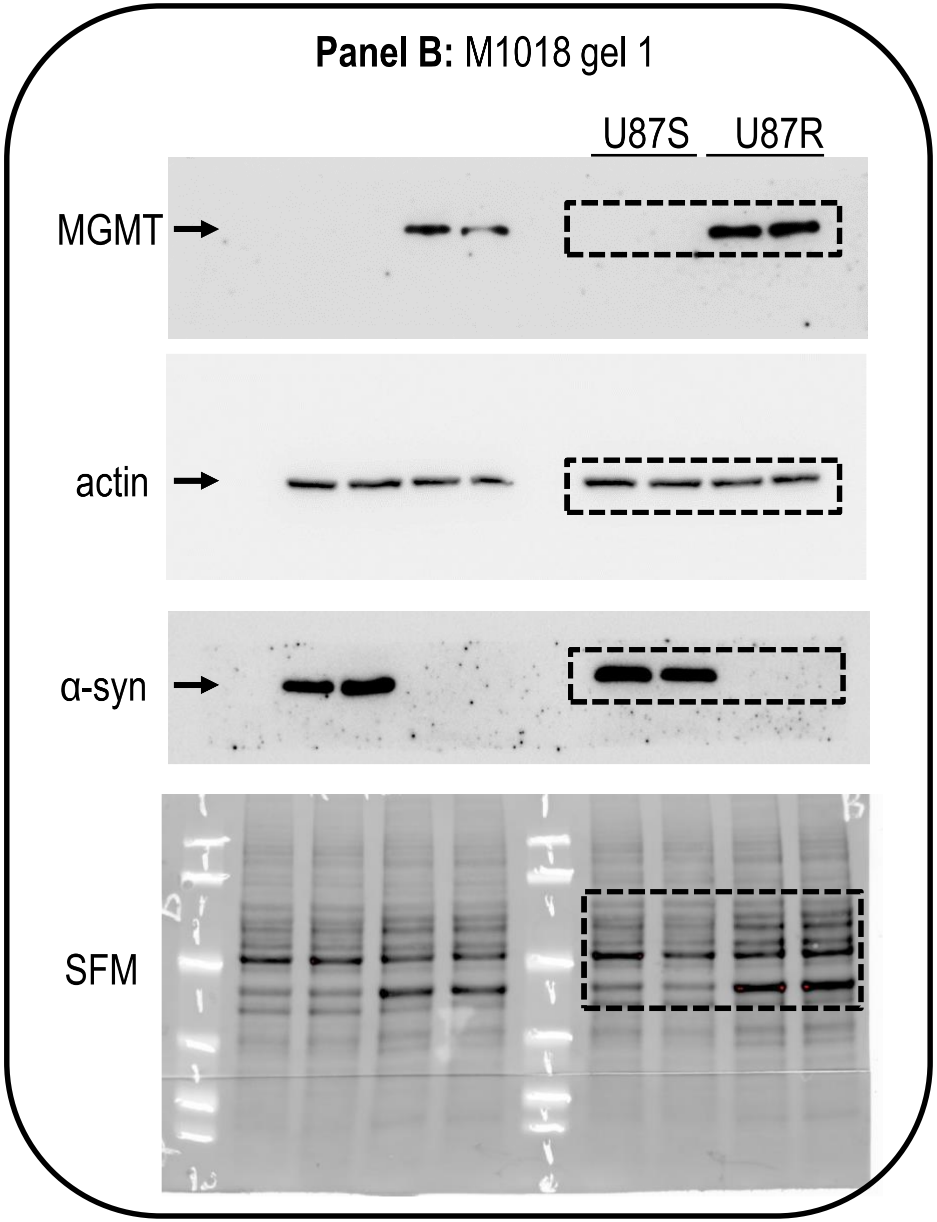

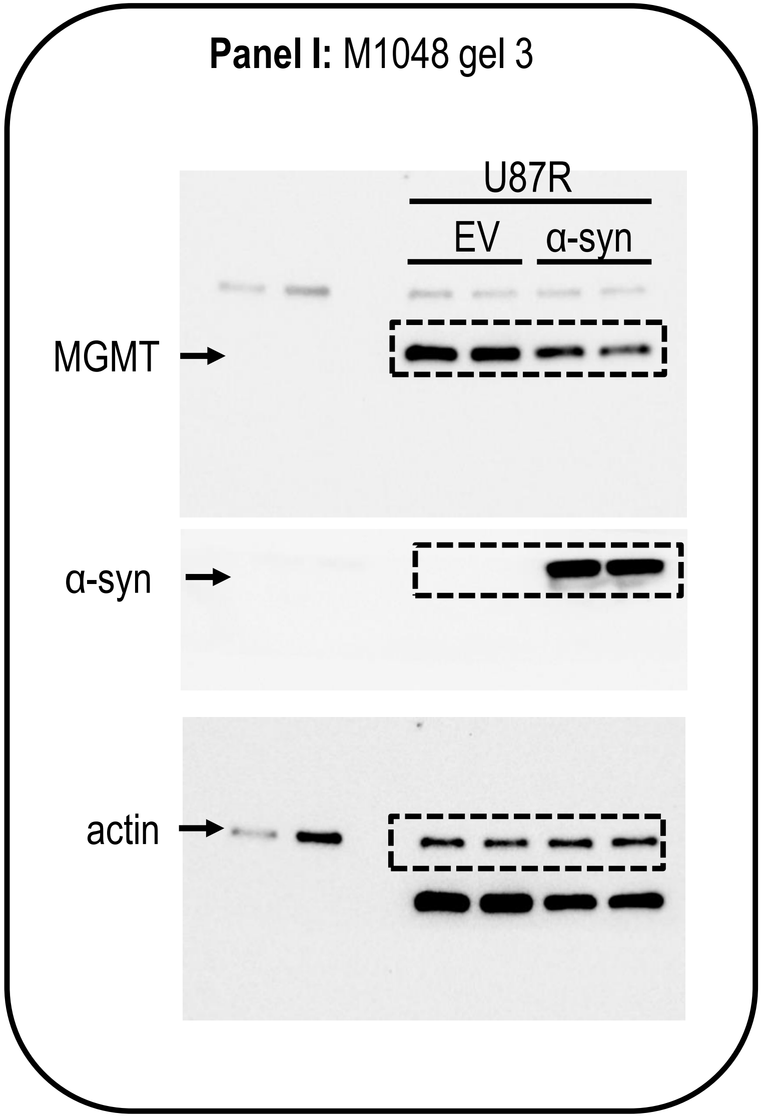

Gels for figure 5

Panel A: M1748 gel 3

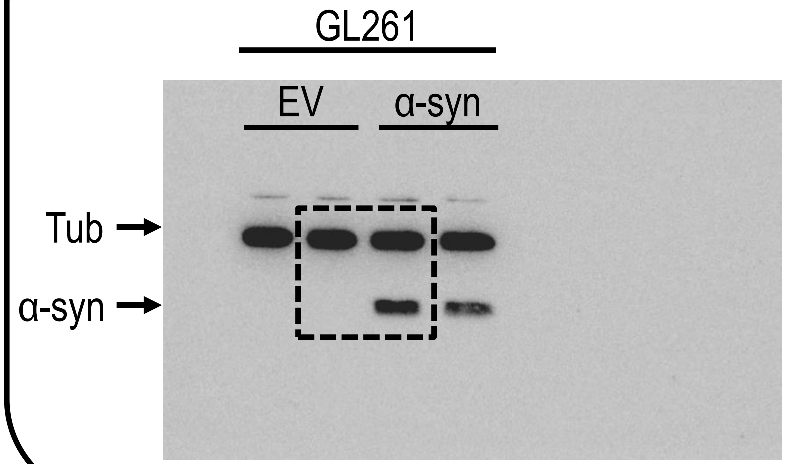

Panel E: M1754 gel 3

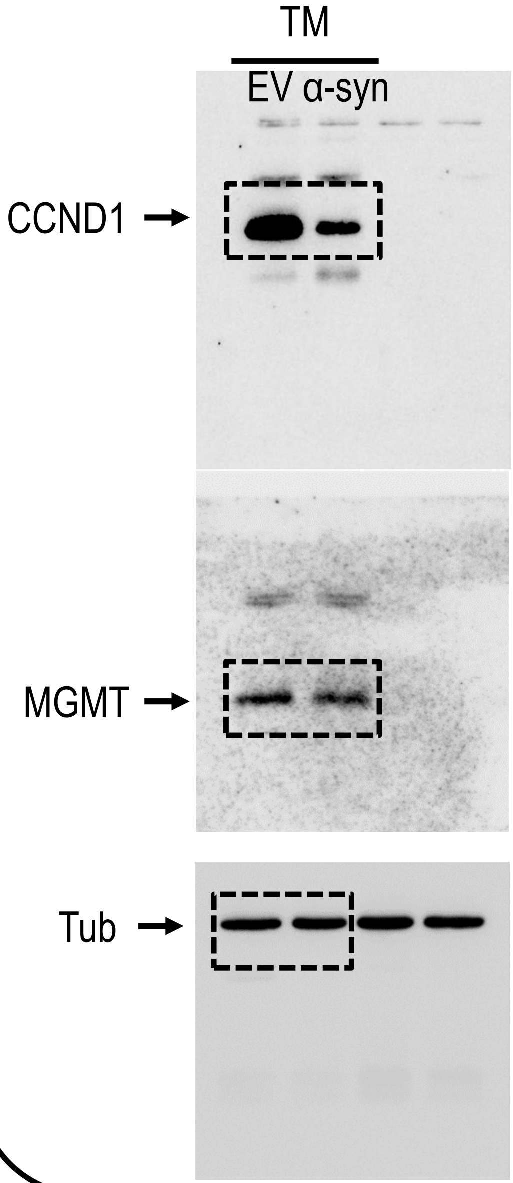

Gels for figure 6

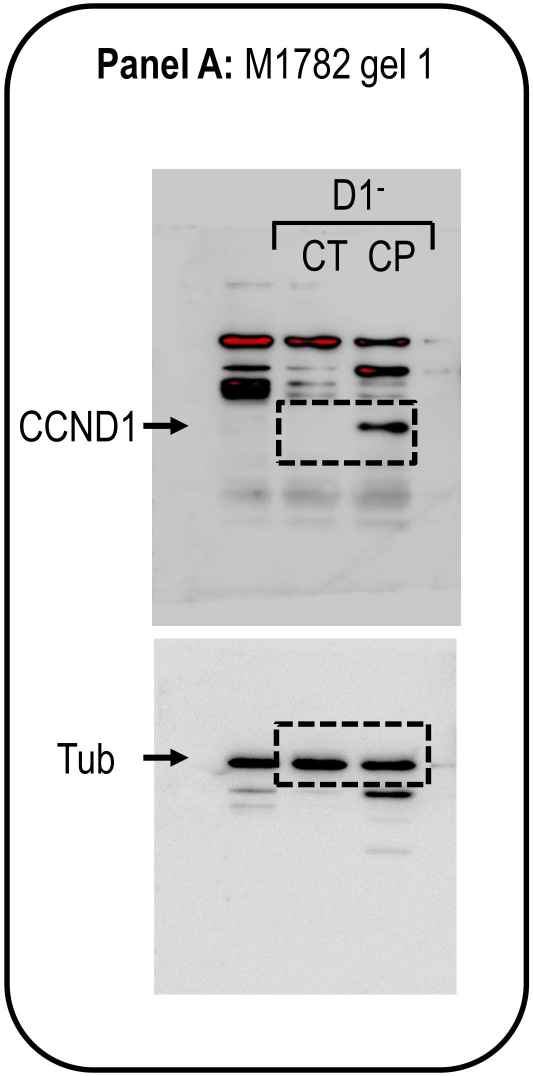

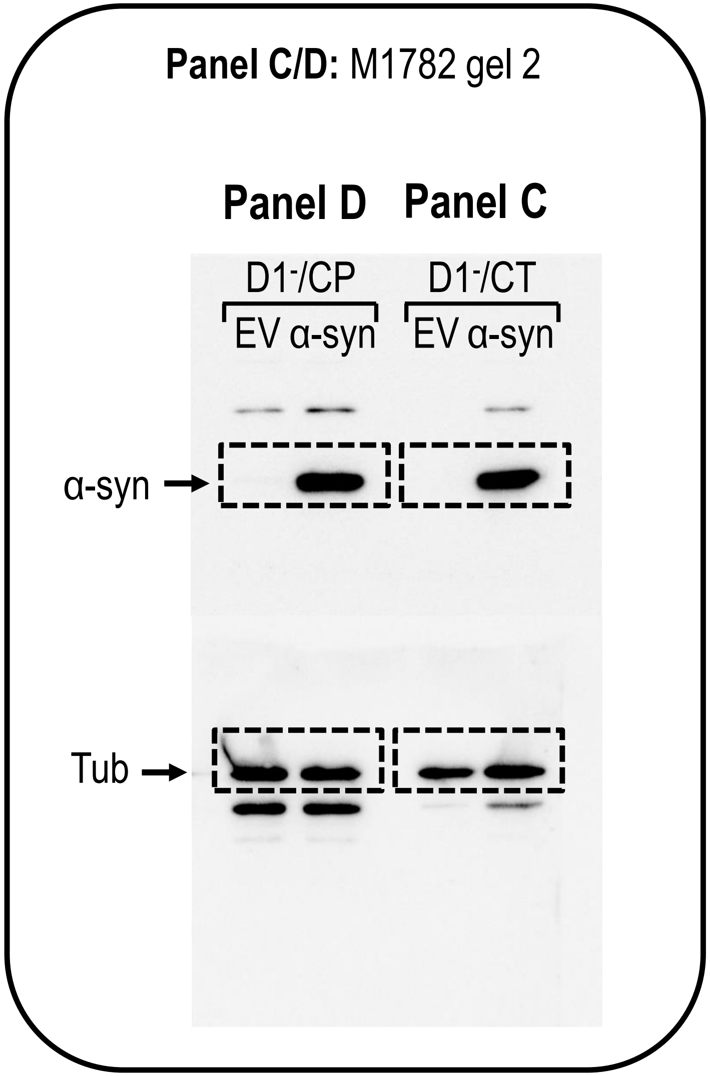

Gels for figure S4

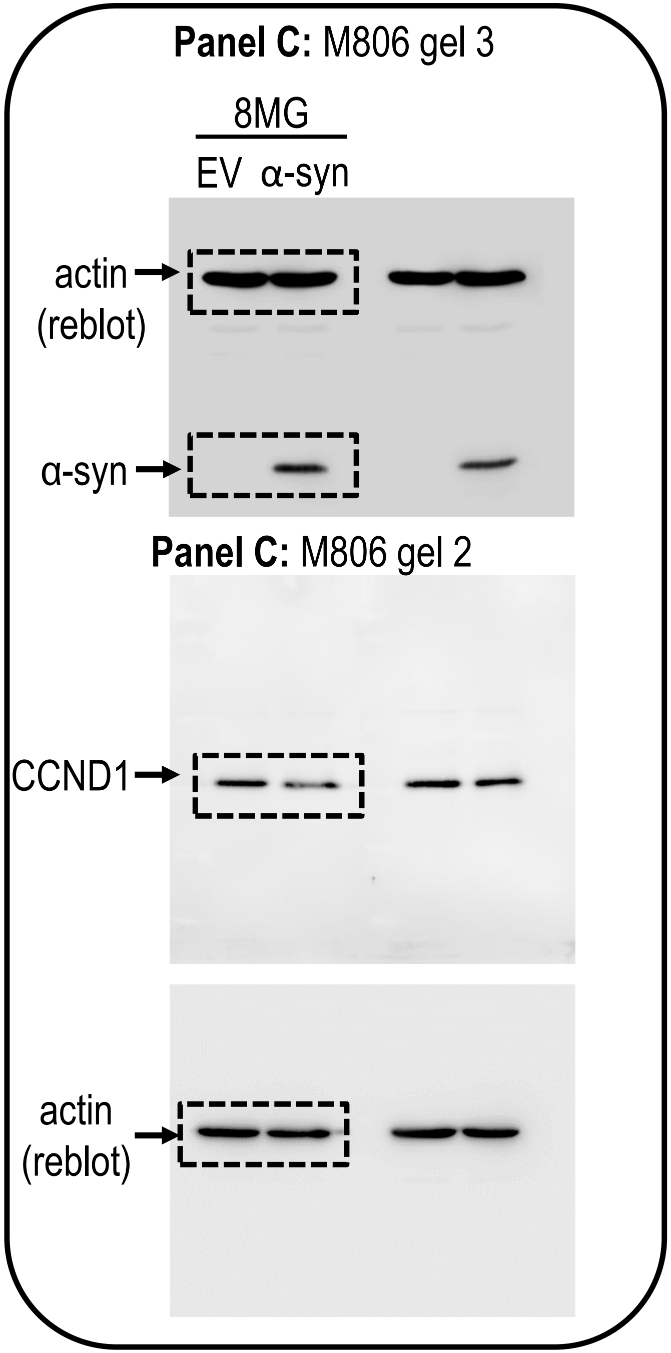

Gels for figure S5

M1774 gel 3

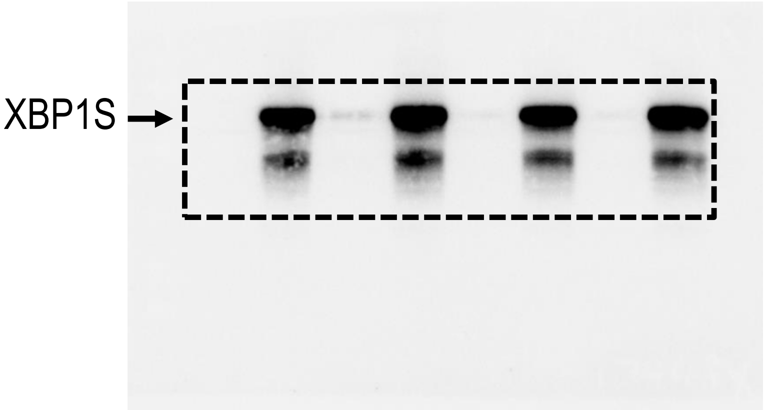

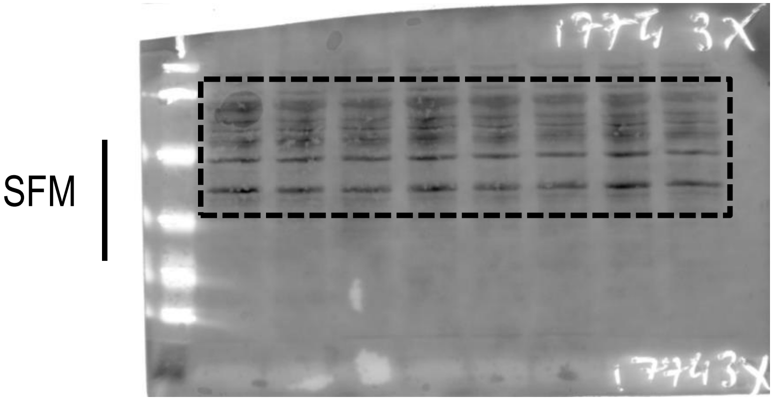

Gels for figure S6

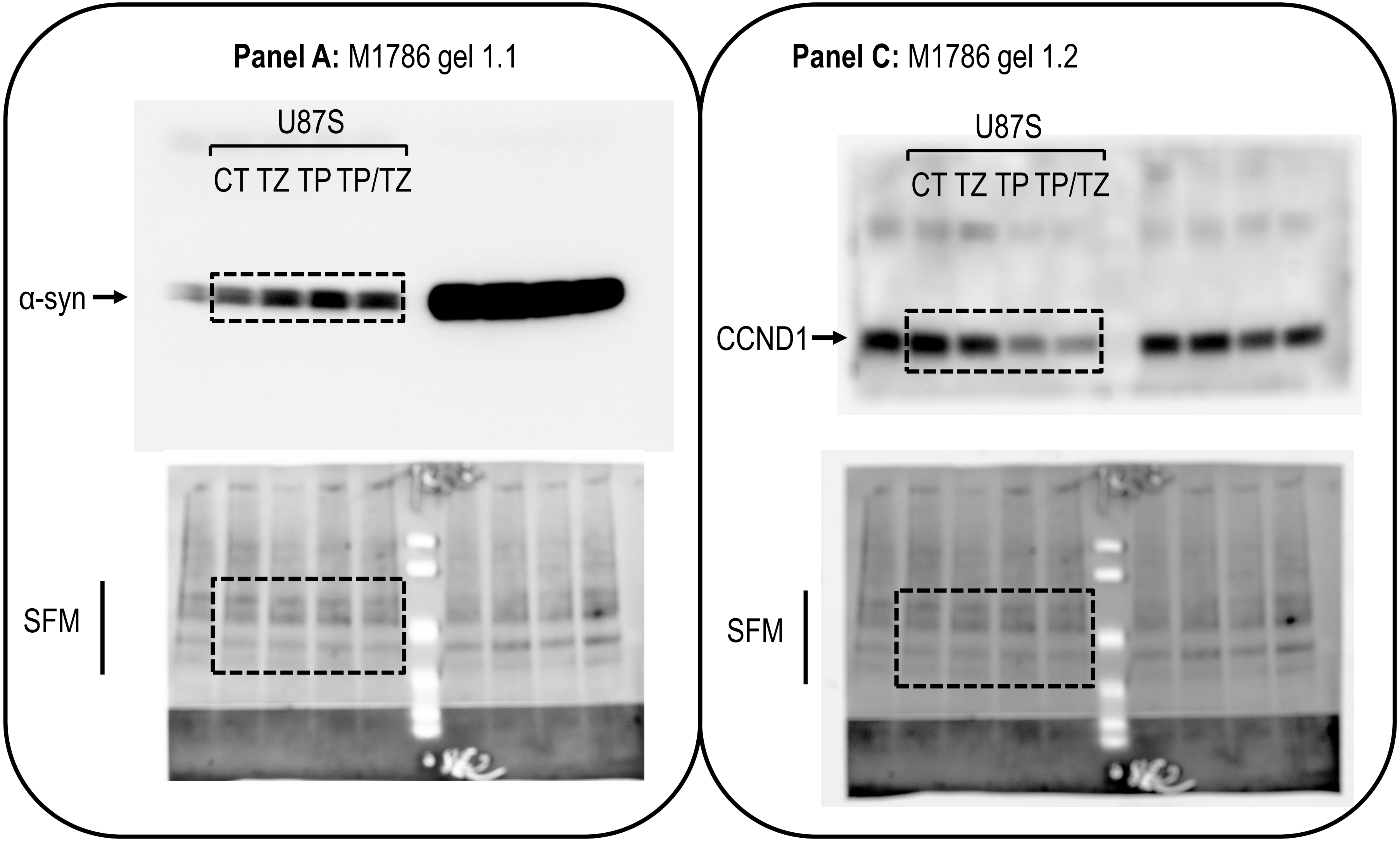

Gels for figure S7
